# Peripheral nerve injury alters metabolic function and neuronal excitability through the Lin28/let-7 pathway

**DOI:** 10.1126/sciadv.aeh6791

**Published:** 2026-10-16

**Authors:** Xinbei Li, Sang-Min Jeon, Ariella Kornfeld, Emily A. Eiss, Daniel S. Jin, Uche Onuchukwu, Manasi Inamdar, Suyeon Kim, Sreenivas Eadara, Matthew Russell, John Robinson, Michael J. Caterina, Mollie K. Meffert

**Affiliations:** ^1^ Solomon H. Snyder Department of Neuroscience, Johns Hopkins University School of Medicine, Baltimore, MD 21205, USA.; ^2^ Department of Neurosurgery and Neurosurgery Pain Research Institute, Johns Hopkins University School of Medicine, Baltimore, MD 21205, USA.; ^3^ Department of Molecular Biology and Genetics, Johns Hopkins University School of Medicine, Baltimore, MD 21205, USA.

## Abstract

Sensory neurons require efficient metabolic processes to reliably transmit information including touch, temperature, and noxious stimuli from peripheral to central nervous systems. Peripheral nerve injury is known to disrupt metabolic function resulting in aberrant sensory nervous system excitation, but molecular pathways linking nerve injury to persistent alterations in metabolism and neuronal activity have been incompletely understood. We find that a conserved metabolic regulator, RNA binding protein Lin28, is elevated in the somas of dorsal root ganglion (DRG) nociceptive neurons following nerve injury but exhibits a higher and more enduring local induction in peripheral sensory axons of the injured nerve. Growth suppressor let-7 family microRNAs (miRNAs), whose biogenesis is inhibited by Lin28, are down-regulated following injury, consistent with the spatiotemporal course of Lin28 induction. Genome-wide sensory neuron-specific and nociceptor-enriched profiling of miRNA:target RNA interactions reveals abundant let-7 miRNA targeting of metabolic transcripts in healthy sensory axons, which is diminished by nerve injury. Consistently, injured sciatic nerves exhibit disrupted cellular respiration near the site of nerve injury, which is alleviated by local restoration of let-7 miRNAs. Selective Lin28 knockout in nociceptive sensory neurons also alleviates the metabolic disturbances of nerve injury as well as suppressing injury-induced elevation of activity in ipsilateral peripheral sensory neurons and central spinal cord dorsal horn neurons. These results implicate the Lin28/let-7 pathway as a critical driver of metabolic dysregulation and the pathophysiology of heightened sensory nervous system activity following nerve injury.

## INTRODUCTION

Peripheral nerve injury with damage to sensory neurons, including nociceptors specialized to detect noxious stimuli, can lead to lesion-induced changes in the bioenergetic and sensory functions of both injured neurons and neighboring, uninjured neurons. Robust protein synthesis occurs within both the cell bodies and axonal processes of peripheral sensory neurons and is observed to increase following peripheral nerve injury, particularly in axons. Inhibiting nascent protein synthesis within afferents impairs neuroplastic responses to injury, including associated alterations in axonal growth, growth cone structure (*1*, *2*), and metabolism (*3*–*5*). Although basal protein synthesis contributes to axonal maintenance and viability, the altered transcript specificity of mRNA translation following nerve injury critically determines protein output and functional outcomes such as regeneration and sensory hypersensitivity. Mitogen-activated protein kinase/extracellular signal–regulated kinase (MAPK/ERK) activation and downstream alterations in protein synthesis present a well-characterized signaling mechanism that contributes to enduring changes in nociceptive responses following nerve injury (*6*–*10*). In previous studies, our lab and others found that MAPK pathway activation can induce the RNA binding protein, Lin28 (*11*–*15*), which inhibits maturation of *lethal-7* (let-7) microRNA (miRNA) precursors into mature functional miRNAs. Vertebrates have two Lin28 paralog genes (Lin28a and Lin28b), which both suppress let-7 miRNA biogenesis and are reciprocally regulated by let-7. Both Lin28 paralogs act as gatekeeper molecules in growth-related signaling pathways and metabolic reprogramming in multiple tissues and cells (*11*, *16*–*22*).

Let-7 miRNAs are a family of small noncoding RNAs (sncRNAs) with both broad conservation across animal species (*23*, *24*) and essential growth-regulatory roles in developmental timing, tumor suppression, and metabolism. Many pro-growth mRNAs harbor evolutionarily conserved repressive binding sites for let-7 miRNAs, which are often termed “growth suppressor” miRNAs. Correspondingly, decreasing let-7 miRNA levels can derepress pro-growth target transcripts to regulate development, differentiation, tissue repair, plasticity, and other context-dependent responses (*15*, *25*–*28*). Regulation of gene expression by let-7 miRNAs affects cellular bioenergetics at multiple levels, including notably through the regulation of glucose metabolism and insulin signaling pathways by let-7–targeted genes associated with metabolic reprogramming and linked to type 2 diabetes mellitus (*29*–*31*). Deficiency of Lin28, and consequent elevation of let-7 miRNAs, alters cellular bioenergetics to produce lowered adenosine triphosphate/adenosine diphosphate (ATP/ADP) and reduced/oxidized nicotinamide adenine dinucleotide (NADH/NAD+) ratios (*17*).

The role of let-7 miRNAs in cellular metabolic reprogramming from glycolysis to mitochondrial oxidative phosphorylation (OXPHOS) as a predominant energy source has been established in several biological contexts including oncogenesis and immune cell activation (*32*–*34*). In sensory neurons, mitochondrial dysfunction plays a prominent role in producing hyperactivity at later time points following injury (*35*–*37*). Elevated cellular oxygen consumption and a disrupted proton gradient across the mitochondrial inner membrane have also been observed in several forms of neuropathy (*35*, *38*, *39*). Adequate regulation of mitochondrial energy production by OXPHOS in peripheral sensory neurons has been posited as a requirement for the resolution of hypersensitivity following peripheral nerve injury (*40*). In addition, neuropathic pain is commonly observed in heritable diseases resulting from mitochondrial mutations (*41*), and dysregulated cellular respiratory metabolism has been proposed as a contributing factor to altered sensory processing, including hypersensitivity (*42*). Despite these insights, molecular mechanisms that might mediate the regulation of metabolism following nerve injury remain poorly characterized.

In this study, we found that peripheral nerve injury produces a sustained local elevation in Lin28 RNA binding protein transcripts and a corresponding sustained reduction in let-7 family miRNA levels. This finding prompted investigation of the critical pathways targeted by miRNAs following nerve injury using a discovery-based strategy, CIMERA-seq (cross-linking and immunoprecipitation followed by intermolecular ligation of endogenous RNAs bound to Argonaute and high-throughput sequencing), for cell-type–selective genome-wide profiling of miRNA:target RNA interactions that functions by ligation of miRNA to target transcripts and RNA sequencing (RNA-seq) of the resulting chimeric RNAs (*43*–*46*). Interactions between let-7 miRNAs and metabolic target genes were abundant in sensory neuron afferents, targeted using the Nav1.8 promoter, and were depleted following nerve injury. Concordantly, altered metabolic activity was observed in injured peripheral nerves, which was alleviated by local restoration of let-7 miRNAs to injured nerves or by conditional loss of Lin28 in Nav1.8 lineage sensory neurons, revealing both spatial and cell-type–specific components of the response to nerve injury. Further, the selective loss of Lin28 in peripheral sensory neurons suppressed injury-induced elevated activity in both neighboring, spared peripheral sensory neurons and ipsilateral spinal cord dorsal horn neurons. Collectively, these findings highlight a mechanism for local Lin28/let-7 pathway induction in peripheral axons of injured sensory neurons to regulate metabolism and sensory excitability and implicate local modulation of the Lin28/let-7 pathway as a potential future strategy to target sensory neuron hyperactivity following nerve injury.

## RESULTS

### Nerve injury induces the Lin28/let-7 miRNA pathway in sensory neurons

Down-regulation of let-7 miRNAs is observed in settings of tissue injury in mammalian systems, whereas Lin28 up-regulation or overexpression has been reported to support recovery under conditions of optic nerve or spinal cord injury and in myocardial or cerebral ischemia (*47*, *48*). In myocardial injury, reported protective effects of Lin28 induction have included enhancing survival by affecting mitochondrial and metabolic function (*49*–*51*). Increased understanding of molecular mechanisms by which the Lin28/let-7 pathway acts following nerve injury has the potential to shed light on therapeutic targets. To investigate Lin28/let-7 pathway responses in peripheral nerve injury, we used both sciatic nerve transection (SNT) and spared nerve injury (SNI) models. SNT involves complete transection of the sciatic nerve and can achieve uniform axonal injury across all branches (*52*, *53*), whereas SNI involves selective transection of the peroneal and tibial branches of the mouse sciatic nerve but spares the sural branch. The SNI partial injury model leads to hyperexcitability in both the spared sensory neurons associated with the intact sural branch and their central spinal targets, effectively mimicking the clinical hypersensitivity observed following partial nerve damage (*54*, *55*). We first investigated whether nerve injury altered levels of Lin28 RNA binding protein transcripts in the mouse dorsal root ganglion (DRG), which houses the cell bodies of peripheral sensory neurons. Digital polymerase chain reaction (PCR) was performed for Lin28 transcripts in mouse lumbar DRG lysates, collected at baseline (naive) and at 3, 7, and 28 days following SNT surgery. We observed an ∼2-fold increase in both Lin28a and Lin28b mRNAs, the two mammalian paralogs, in the injured DRGs ipsilateral to SNT, compared to contralateral uninjured DRGs at 3 days postinjury (Fig. 1, A and B). However, significant differences in Lin28 were no longer detected at 7 or 28 days postinjury (Fig. 1, A and B).

**Fig. 1.**
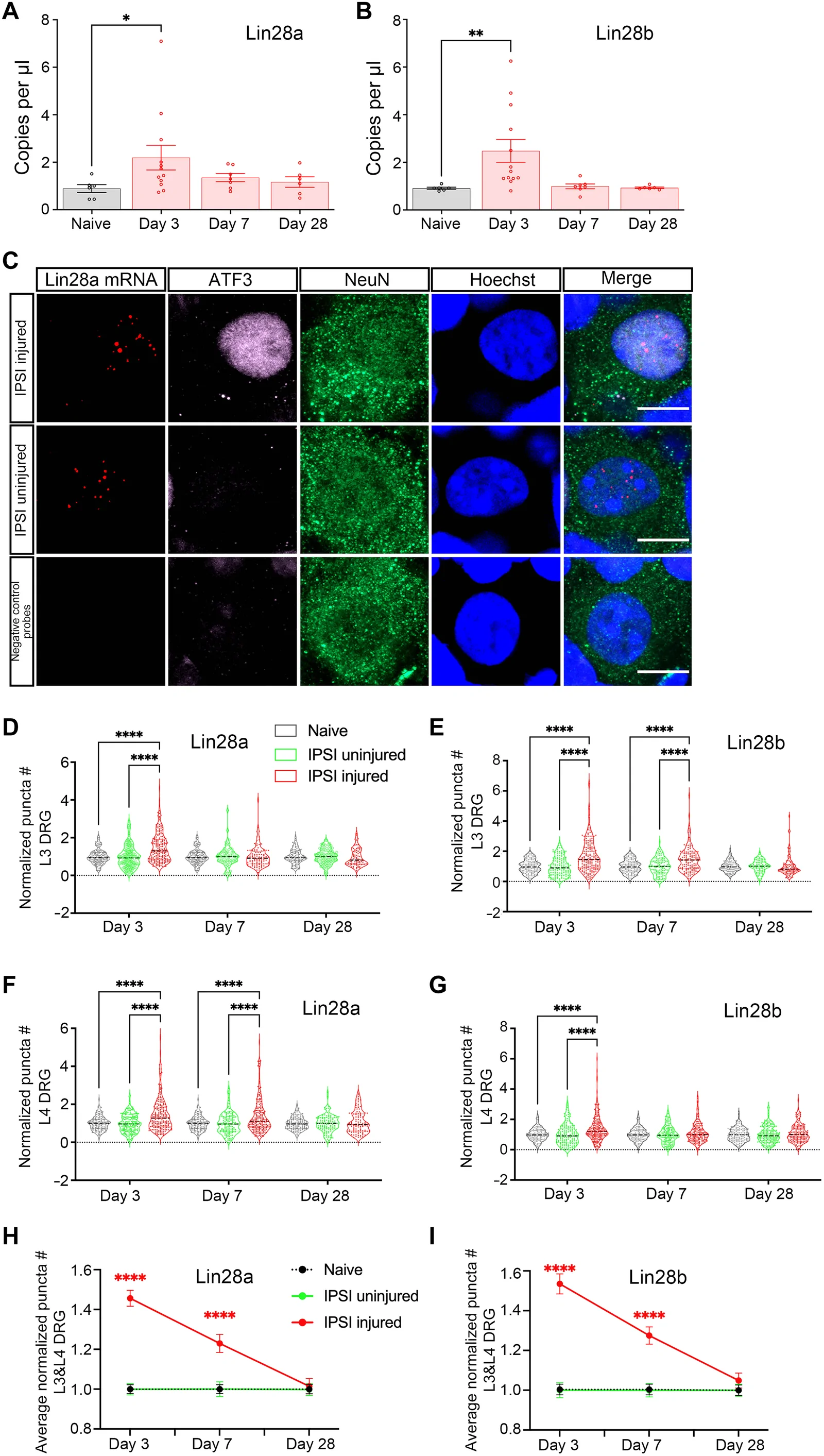
Nerve injury affects Lin28 abundance in sensory neuron DRGs. (**A** and **B**) Digital PCR for Lin28a (A) and Lin28b (B) fold gene expression in DRGs at 3, 7, and 28 days postinjury (SNT), normalized to contralateral average (set as 1.0) at each time point. Two-tailed unpaired Student’s *t* test with Dunnett’s; *N* = naive (12), day 3 (13), day 7 (7), and day 28 (8). (**C**) Representative Lin28a mRNA RNAscope in orthogonal projections from confocal *Z* stacks in ipsilateral injured and uninjured DRG neurons at 3 days postinjury (SNI), with neurons (NeuN), injured neurons (ATF3), and nuclei (Hoechst). Scale bars, 10 μm. (**D** and **E**) Lin28a (D) and Lin28b (E) mRNA puncta number in ipsilateral and naive L3 DRG small- to medium-sized neurons at 3, 7, and 28 days postinjury (SNI); normalization yokes injured to uninjured neuron average (set as 1.0) within an image; naive neurons normalized to naive neurons average (set as 1.0). *N* = 250 to 350 cells from seven male mice per condition. (**F** and **G**) Lin28a (F) and Lin28b (G) mRNA puncta quantification in ipsilateral and naive L4 DRG small- to medium-sized neurons at 3, 7, and 28 days postinjury (SNI); normalization yokes injured to uninjured neurons average (set as 1.0) within an image; naive neurons normalized to naive neurons average (set as 1.0). *N* = 250 to 350 cells from seven male mice per condition. (**H** and **I**) Averaged Lin28a (H) and Lin28b (I) mRNA puncta in ipsilateral and naive L3 to L4 DRG small- to medium-sized neurons at 3, 7, and 28 days postinjury (SNI); normalization yokes injured to uninjured neurons average (set as 1.0) within an image; naive neurons normalized to naive neurons average (set as 1.0). *N* = 7 male mice per condition. [(C) to (I)]: two-way ANOVA, Tukey’s multiple comparisons. Error bars represent the SEM; *P < 0.05, **P < 0.01, and ****P < 0.0001.

To determine the DRG cell type responsible for the up-regulated Lin28 expression, we mapped the spatiotemporal distribution of Lin28 mRNA using single-molecule in situ hybridization in DRGs collected at baseline and at 3, 7, and 28 days following SNI surgery (*56*). Concurrent immunohistochemical (IHC) staining was used to identify all neurons (NeuN) and injured neurons (ATF3) (Fig. 1C and fig. S1A). The use of SNI, rather than SNT, allowed attribution of Lin28 induction to either injured or uninjured neurons, as well as allowing injured and uninjured neurons to be compared in a yoked manner within the same side of injury to minimize potential confounding contralateral effects. Soma size was used to broadly classify somatosensory neuron subtypes in DRGs according to previously established criteria. Large diameter DRG neurons typically convey proprioception and responses to innocuous mechanical stimulation, such as light touch. DRG neurons with small- to medium-diameter cell bodies, while also including low-threshold mechanoreceptors, consist predominantly of nociceptors or pruriceptors, neurons responsive to noxious or itch-producing stimuli (*57*–*60*). In the small- to medium-diameter sensory neuron population, Lin28a and Lin28b mRNA levels were significantly elevated in injured neurons (NeuN+/ATF3+) compared to neighboring uninjured neurons (NeuN+/ATF3−) or neurons in naive mice at 3 days postinjury in both lumbar 3 (L3) (Fig. 1, D and E, and fig. S1, B and C) and L4 DRG (Fig. 1, F and G, and fig. S1, D and E). Lin28a and Lin28b remained modestly elevated in injured neurons at 7 days postinjury, with statistical significance confined to L4 DRG for Lin28a and L3 DRG for Lin28b. By 28 days post-SNI, the longest time point tested, Lin28a and Lin28b mRNA levels had returned to baseline and were indistinguishable between injured, neighboring uninjured, and naive small- to medium-diameter sensory neuron cell bodies in the DRG. Overall, detectable Lin28 induction in these sensory neuron somas peaked at 3 days postinjury and progressively declined over the time course (Fig. 1, H and I).

### Prolonged Lin28 induction and lowered let-7 miRNA levels in injured nerve axons

Local translation in neuronal processes, including axons of sensory neurons, is crucial for precise spatiotemporal responses to external signals. A subset of mRNAs transcribed in the nucleus is transported to distal neuronal processes, where localized and selective translation plays key roles in neuronal plasticity and the response to nerve injury (*8*, *10*, *61*–*65*). To assess whether Lin28 RNA binding protein transcripts are present in the axons of sensory neurons and whether their abundance changes following nerve injury, we mapped Lin28 mRNAs in the sciatic nerve at baseline and at 3, 7, and 28 days following SNI (*56*). We divided 1-cm injured sciatic nerve segments (containing both tibial and common peroneal components) into three anatomical regions: injured stump, injured middle, and injured proximal (Fig. 2A). The stump region corresponds to the nerve segment nearest the injury site, the proximal region is the segment closest to the DRG (farthest from the injury site), and the middle region lies between the stump and proximal regions. Using single-molecule in situ hybridization combined with IHC staining on mouse sciatic nerves (Fig. 2B), Lin28a and Lin28b mRNA levels were observed to elevate in the injured sensory neuron axons (MAP2+) beginning at 3 days postinjury, with a maximum observed at 7 days postinjury in axons at the nerve stump. This Lin28 elevation persisted at least 28 days following SNI (Fig. 2, C and D), the latest time point tested. The uninjured ipsilateral sural nerve axons also exhibited elevated Lin28 mRNA levels at 3 and 7 days postinjury, although statistical significance was not reached for 28 days postinjury, possibly due to greater variability (fig. S2, A to C). Lin28a and Lin28b were also elevated in Schwann cells (S100+) within the injured nerve and the uninjured sural nerve throughout the post-SNI time course but to a lesser extent than in axons (fig. S2, D and E). In contrast, abundance of a control transcript, peptidylprolyl isomerase B (Ppib), remained unchanged following nerve injury (Fig. 2E and fig. S2, C and F). We have previously demonstrated that we can reliably distinguish axonal and Schwann cell localization of RNA puncta with our imaging and analysis protocols (*56*). Notably, the elevation of Lin28 transcripts exhibited both a more enduring induction and a greater amplitude in injured peripheral sensory axons compared to the somatic compartment of these sensory neurons following nerve injury (Fig. 2F). These intriguing findings indicated pronounced gene regulatory differences between the cell bodies and axons of sensory neurons, as well as revealing a persistent change in axonal Lin28 consistent with the chronicity of responses to nerve injury.

**Fig. 2.**
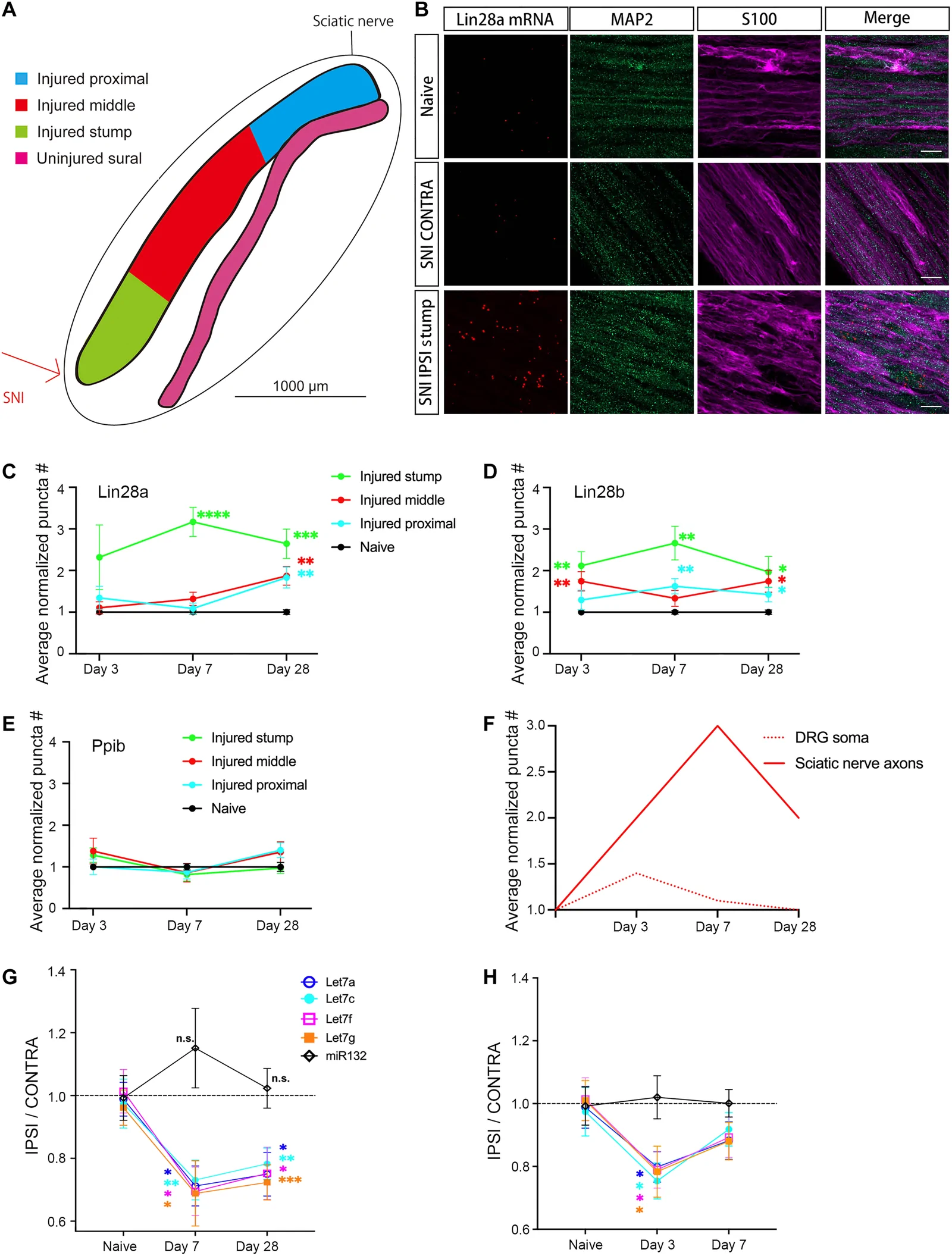
Prolonged Lin28 induction and lowered let-7 miRNA levels in axons of injured nerve. (**A**) Diagram of ipsilateral injured sciatic nerve anatomical ROIs. (**B**) Representative Lin28a mRNA RNAscope staining of the nerve stump at 7 days postinjury (SNI) in the naive sciatic nerve, contralateral uninjured sciatic nerve, and ipsilateral injured sciatic nerve. Neuronal processes are labeled with MAP2, Schwann cells are labeled with S100. Images are orthogonal projections from *Z*-stack confocal images. Scale bars, 10 μm. (**C** to **E**) Quantification of Lin28a (C), Lin28b (D), and Ppib (E; positive control) mRNA puncta in naive and ipsilateral neuronal processes (MAP2+) at 3, 7, and 28 days postinjury (SNI). The number of mRNA puncta in ipsilateral injured nerves is normalized to contralateral uninjured nerves (set as 1.0) at each time point. The number of mRNA puncta in naive nerves is normalized to naive nerves average (set as 1.0). Statistical comparisons based on ROI; *N* = 40 to 60 from seven male mice per condition. (**F**) Lin28a mRNA normalized mRNA puncta in injured DRG neurons and sciatic nerves at 3, 7, and 28 days postinjury. (**G**) Digital PCR data for let-7 family miRNAs (let-7a, let-7c, let-7f, and let-7 g) and negative control miR-132 FGE in sciatic nerves at 7 and 28 days postinjury (SNT); expression normalized to contralateral average (set as 1.0) at each time point. *N* = naive (6), day 7 (6), and day 28 (7) per condition. (**H**) Digital PCR data for let-7 family miRNAs (let-7a, let-7c, let-7f, and let-7 g) and negative control miR-132 FGE in DRGs at 3 and 7 days postinjury (SNT). Expression levels are normalized to contralateral side average (set as 1.0) at each time point. *N* = naive (6), day 7 (6), and day 28 (7) per condition. Statistical comparisons [(B) to (H)]: two-way ANOVA; Tukey’s multiple comparisons test. Error bars represent the SEM; *P < 0.05, **P < 0.01, ***P < 0.001, and ****P < 0.0001; n.s., not significant.

Because Lin28 RNA binding proteins negatively regulate let-7 miRNAs by inhibiting their biogenesis, we evaluated whether mature let-7 miRNA levels were also affected by the injury-induced Lin28 up-regulation. We observed a persistent reduction in all tested let-7 family miRNA members by digital PCR, including let-7a, let-7c, let-7f, and let-7g, within the injured sciatic nerve (SNT), correlating with the spatiotemporal pattern of Lin28 induction, whereas a control miRNA miR-132 showed no significant change (Fig. 2G). In contrast, let-7 miRNA levels assayed by digital PCR in DRGs, containing sensory neuron somas, were not significantly reduced ipsilateral to the injury beyond 3 days post-SNT (Fig. 2H). These data are consistent with elevation of both Lin28 RNA and Lin28 protein, which regulates let-7 miRNAs. The prolonged Lin28 elevation in sensory neuron axons suggested the possibility that Lin28, along with its regulation of let-7 family miRNAs, might play a localized role in distal neuronal processes in prolonged responses to nerve injury.

### Sensory neuron-selective miRNA:target RNA interaction profiling reveals let-7 miRNA targeting of metabolic genes

MiRNAs in peripheral nerves can coordinate gene expression, affecting a broad range of known physiological responses to nerve injury including metabolic regulation, neuronal survival and regeneration, and modulation of ion channel expression and neuronal excitability (*10*). To investigate changes in targeting of transcripts by altered let-7 miRNA levels in response to nerve injury, we considered bioinformatic tools, which can predict potential miRNA:target RNA interactions, but these tools do not report consequences of altered miRNA levels on target gene repression and yield only a theoretical target output which can be confounded by biological variables affecting miRNA interactions and lack quantitative capacity. To overcome these limitations, we used an optimized technique, CIMERA-seq, to directly profile in vivo genome-wide miRNA:target RNA interactions (*43*–*46*). In CIMERA-seq, miRNAs are ligated to their target RNAs to form chimeric RNAs while bound to the Argonaute (Ago) protein of the RNA-induced silencing complex (RISC) (Fig. 3A). Following isolation and immunopurification, chimeric miRNA:target RNA libraries are constructed for high-throughput sequencing. We engineered a sensory neuron cell-type–selective application of CIMERA-seq (*43*) by crossing a mouse line harboring a conditionally expressed tagged form of Ago [Lox-STOP-Lox (LSL)-GFP-myc-Ago2 allele] with Nav1.8-Cre mice (*66*–*69*), in which Cre recombinase is expressed downstream of the Nav1.8 promoter in a subpopulation of sensory neurons that includes most nociceptors (Fig. 3B and fig. S3, A and B). This strategy allows selective expression of the tagged Ago proteins in Nav1.8 lineage sensory neurons, enabling cell-type–selective genome-wide profiling of miRNA:target RNA interactions. MiRNA:target RNA-seq data were analyzed using SCRAP (*44*, *45*), a computational pipeline that uses peak calling to stringently identify high confidence, robust miRNA:target RNA interactions for further investigation.

**Fig. 3.**
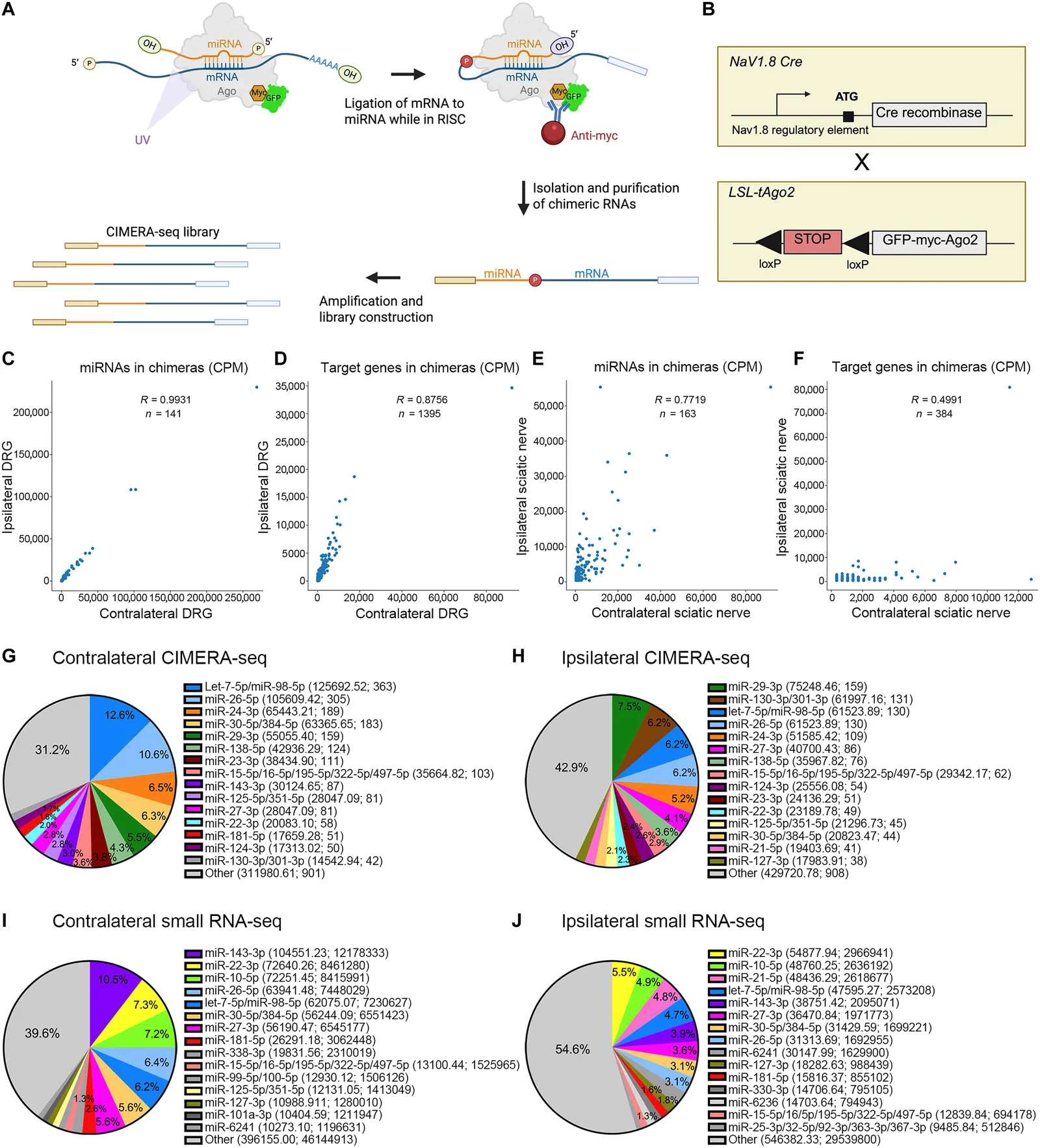
Nociceptor-selective miRNA:target RNA interactions profiling in vivo. (**A**) Nociceptor-selective miRNA:target RNA interaction profiling using CIMERA-seq. RNAs are UV cross-linked to GFP-myc–tagged Argonaute (tAgo2). The 5′ target RNA is ligated to the 3′ miRNA to form a chimeric RNA. Chimeric RNA-tAgo2 complexes are immunoprecipitated using anti-myc, and chimeric RNAs are isolated and library constructed by ligating adapters for PCR amplification and sequencing. (**B**) Cre-Lox system allowing nociceptor-selective expression of tAgo2. (**C** to **F**) Correlation matrices between all miRNA (C and E) and all gene (D and F) counts obtained from nociceptor-selective miRNA:target RNA profiling in contralateral uninjured (*x* axes) and ipsilateral injured (*y* axes) DRG (C and D; 3 days postinjury) or sciatic nerves (E and F; 7 days postinjury). Each dot represents one miRNA (C and E) or gene (D and F) from CIMERA-seq. For DRG, *n* = 141 miRNAs with counts > 0 in both datasets and *n* = 2936 genes with counts > 0 in both datasets, normalized as CPM. For sciatic nerve, *n* = 163 miRNAs with counts > 0 in both datasets and *n* = 384 genes with counts > 0 in both datasets, normalized as CPM. (**G** and **H**) Distribution of miRNAs (individual or families) detected by CIMERA-seq from contralateral (G) or ipsilateral sciatic nerve (H), 7 days postinjury. Top 15 miRNAs/families shown, with the remainder labeled “Other”. Fraction of each miRNA/family in CPM across all samples is depicted; CPM values and raw reads are shown in parentheses. *n* = 2888 (G) or *n* = 2113 (H), deduplicated chimeric reads. (**I** and **J**) Distribution of miRNAs (individual or families) detected in bulk small RNA-seq data from contralateral (I) or ipsilateral sciatic nerve (J), 7 days postinjury. Top 15 miRNAs/families are shown, with the remainder labeled as “Other.” The fraction of each miRNA/family in CPM across all samples is depicted; CPM values and raw reads are shown in parentheses. *n* = 116,481,970 (I) or *n* = 54,064,364 (J) total sequencing depth.

To broadly evaluate whether nerve injury and consequent altered miRNA levels affected miRNA:target RNA interactions, we first compared the relative ipsilateral and contralateral abundance of miRNAs and target genes detected in high-confidence CIMERA-seq interactions. In the DRG, sensory neuron miRNA:target RNAs in high-confidence interactions showed relatively strong correlation between ipsilateral and contralateral lumbar DRG at 3 days postinjury (SNI) (peak Lin28 induction in sensory neuronal somas) for both miRNAs and targeted transcripts (Fig. 3, C and D, *R* = 0.99, 0.87, respectively). This finding is consistent with modest or restricted injury-induced differences in gene targeting by miRNAs in DRG neuronal cell bodies. In contrast, in the sciatic nerve, which contains sensory axons, high-confidence neuronal miRNA-target RNA interactions showed lower correlations between ipsilateral and contralateral nerves at 7 days postinjury (SNI) (peak Lin28 induction in sensory neuronal axons) (Fig. 3, E and F, *R* = 0.77, 0.49, respectively). In particular, we noted conspicuous differences in CIMERA-seq detected target genes in injured compared to contralateral uninjured sciatic nerve miRNA:target RNA interactions, suggesting functional gene regulation from injury-induced changes in RISC engagement.

Evaluation of the specific miRNAs participating in miRNA:target RNA interactions in sciatic nerves at 7 days postinjury (SNI) revealed an altered profile, which included a notable ipsilateral decrease in let-7 miRNA:target RNA interactions, both in total abundance of interactions as well as in relative proportion to other miRNAs; let-7 family miRNAs participated in 12.6% of all interactions in contralateral nociceptive axons, compared to just 6.2% of all interactions in ipsilateral nociceptive axons (Fig. 3, G and H). Bulk small RNA-seq of sciatic nerves at 7 days postinjury (SNI) correspondingly showed a decrease in total let-7 miRNAs in ipsilateral relative to contralateral nerves, with a two-thirds reduction in raw read counts and a decrease in let-7 miRNA representation from 6.2 to 4.7% of all detected miRNAs (Fig. 3, I and J). The reduction in total let-7 miRNA levels in ipsilateral nerves may be muted relative to the pronounced reduction of let-7 participation in mRNA targeting in part because sciatic nerve bulk small RNA-seq includes cell types additional to the Nav1.8 lineage sensory neuron axons, which are selected in CIMERA-seq, and/or because changes in axonal let-7 miRNA interactions with target RNAs are more pronounced compared to bulk axonal let-7 miRNA changes.

The finding that let-7 miRNA:target RNA interactions underwent the most marked decline in injured nociceptive sensory axons, evident by both percentage and total counts per million (CPM) (Fig. 3, G to J), motivated pathway analyses of the target genes from interactions enriched in contralateral axons relative to the let-7–depleted injured ipsilateral axons. The let-7 miRNA–targeted genes were categorized by Gene Ontology (GO) analysis based on cellular components (CC), molecular function (MF), and biological process (BP) (Fig. 4A and fig. S4A). Consistent with origin from a nerve process, the CC category, reflecting subcellular localization, showed enrichment for terms related to cellular junctions, membrane/cytoskeleton, myelin, and receptors (fig. S4A). In the MF and BP categories, which reflect biochemical activities and biological pathways of gene products, respectively, we noted multiple terms related to metabolism, including metabolic processes and metabolic regulation as well as oxidoreductase, guanosine triphosphatase (GTPase), and adenosine triphosphatase (ATPase) activities (Fig. 4A). Comparison of the gene targets detected by CIMERA-seq (Fig. 4B and table S1) underscored let-7–targeted genes associated with metabolism and glucose utilization for which the gene targeting was lost or diminished in the ipsilateral injured nerve, where let-7 miRNA levels are reduced. This included an overall decline in ipsilateral let-7 miRNA targeting of transcripts encoded by the mitochondrial genome (Fig. 4B, shown in red). Canonical miRNA-target RNA interactions classically involve base pairing of miRNA “seed” sequence regions (miRNA nucleotide positions 2 to 8), which are homologous across let-7 miRNA family members (*70*–*72*). Consistent with this pattern, examples of detected let-7 miRNA binding interactions (plcb1, pdk2, zfhx3, mt-Nd1, mt-Nd2, and mt-Cytb) observed in our data (Fig. 4C) correspondingly illustrate complementary base pairing with the miRNA seed region, located at the miRNA 5′ end.

**Fig. 4.**
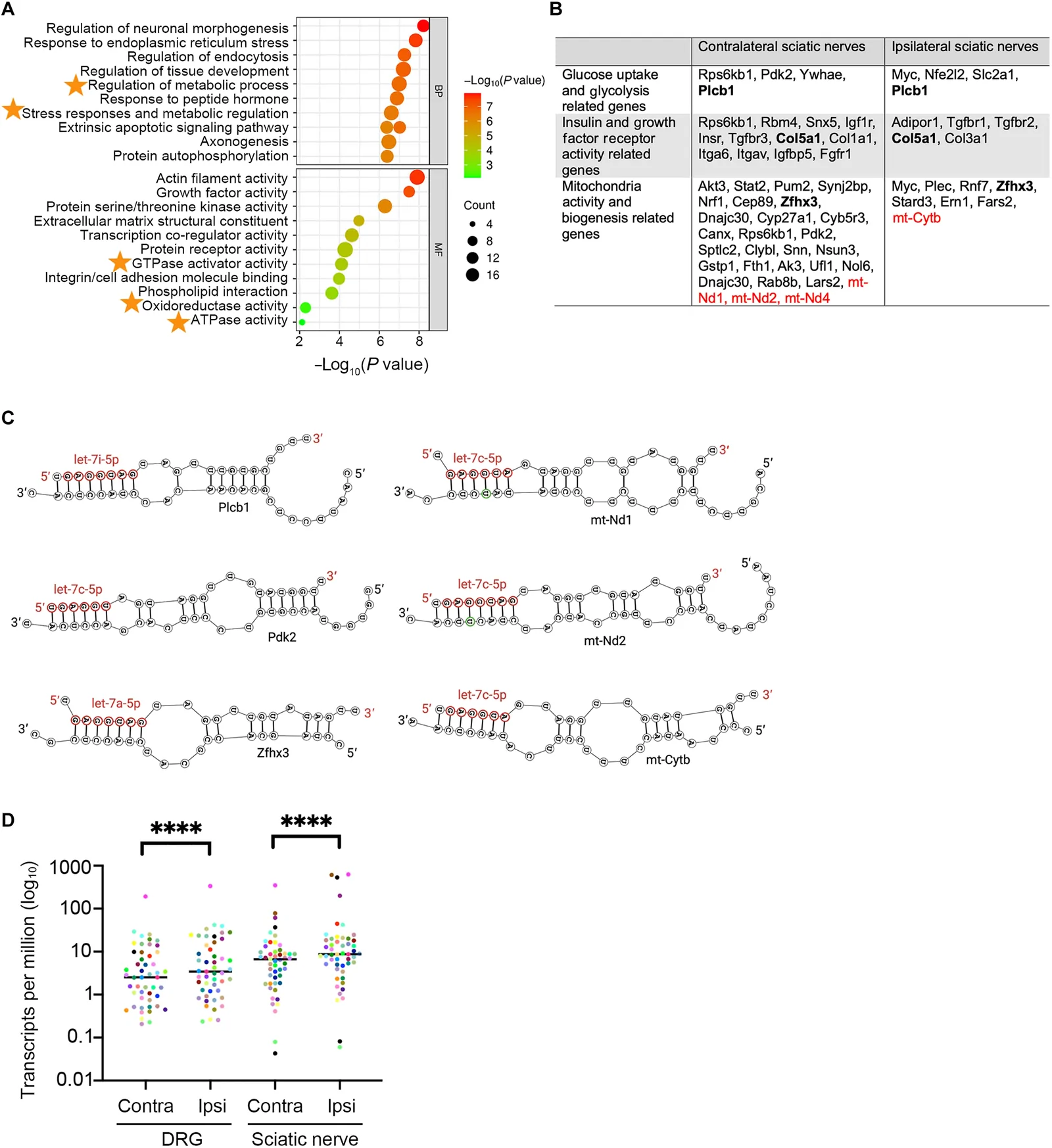
Nociceptor-selective miRNA:target RNA interactions profiling reveals let-7 miRNA targeting on metabolic genes. (**A**) GO enrichment analysis for BP and MF presenting the top 10 GO terms for let-7 miRNA gene targets enriched in contralateral sciatic nerve CIMERA-seq compared to ipsilateral sciatic nerve CIMERA-seq at 7 days postinjury. The *x* axis is enrichment score [−log_10_(*P* value)]; dot size represents the number of genes; color bar indicates the *P* value. Metabolic related terms are highlighted with stars. (**B**) Let-7–targeted metabolic genes detected in contralateral sciatic nerve CIMERA-seq and ipsilateral (lowered let-7 miRNA levels) sciatic nerve CIMERA-seq at 7 days postinjury. Transcripts encoded by the mitochondrial genome are shown in red, and transcripts for which the targeting by let-7 miRNAs was detected in both contralateral and ipsilateral sciatic nerves are shown in bold font. (**C**) Examples of miRNA:target RNA binding interactions are displayed for let-7i-5p and Plcb1, let-7c-5p and Pdk2, let-7a-5p and Zfhx3, let-7c-5p and mt-Nd1, let-7c-5p and mt-Nd2, and let-7c-5p and mt-Cytb. The seed sequence is shown in red, and the G-U wobble is in green. (**D**) Comparison of ipsilateral and contralateral bulk transcript levels for let-7 miRNA–targeted nuclear-encoded metabolic genes detected in CIMERA-seq. Ipsilateral and contralateral transcript levels are displayed using bulk RNA-seq data from DRG (3 days postinjury) and sciatic nerve (7 days postinjury). Shared genes in both datasets are represented using points of the same color. Read depths are as follows: contralateral DRG: 5,342,762; ipsilateral DRG: 6,708,542; contralateral sciatic nerve: 8,762,111; ipsilateral sciatic nerve: 6,376,318. Error bars represent the SEM; ****P < 0.0001.

Effective miRNA-mediated gene repression conventionally results in translational repression and/or transcript degradation, which contribute to functional regulation. Although multiple mechanisms govern transcript levels, relief of miRNA-mediated repression can up-regulate target genes. Accordingly, we performed bulk mRNA sequencing to evaluate transcript abundance in settings of altered let-7 miRNA levels: 3 days postinjury (SNI) ipsilateral DRG relative to contralateral DRG, and 7 days postinjury (SNI) ipsilateral sciatic nerve relative to contralateral uninjured nerves. We evaluated the potential impact of let-7 miRNA targeting specifically on transcript levels of nuclear genome–encoded metabolic genes as categorized in the Mouse Genome Informatics (MGI) Mouse Gene Expression Database. Let-7–targeted metabolic genes, profiled in DRGs or sciatic nerve CIMERA-seq, showed a significant average ipsilateral increase in both the ipsilateral DRG and injured sciatic nerve at respective time points (3 or 7 days postinjury) of lowered let-7 miRNAs, consistent with transcript derepression (Fig. 4D). Read depth was similar across all samples; genes in both DRG and sciatic nerve datasets are represented by symbols of the same color (Fig. 4). Mitochondrial genome–derived gene targets were omitted from this analysis as it remains unclear whether transcript degradation as a consequence of miRNA-mediated targeting is to be expected within mitochondria (*73*). Consistent with the analysis of miRNA-targeted transcripts, GO and Kyoto Encyclopedia of Genes and Genomes (KEGG) assessment of gene pathways from bulk sequencing (fig. S4, B and C) revealed a notable ipsilateral enrichment of transcripts for genes implicated in metabolism, including multiple terms related to mitochondria and mitochondrial functions in glucose and fat metabolism. Together, these data highlighted altered regulation of metabolic genes by let-7 miRNAs following nerve injury and implicated the sciatic nerve compartment as a potential site of highest enduring impact.

### Let-7 miRNA pathway mediates metabolic dysregulation following nerve injury

Collectively, our data implicating metabolism as a key differentially regulated pathway in injured nerves, including the targeting of mitochondrial genes (Fig. 4), prompted us to directly investigate cellular metabolism and mitochondrial respiration in these nerves. We measured oxygen consumption rates (OCRs) in ex vivo sciatic nerves (Fig. 5A); day 7 postinjury (SNI) was selected as the time point for analysis based on maximum Lin28 induction, as well as to avoid a peak of initial inflammatory immune cell infiltration observed by others (*74*–*77*). Spatial mapping of the metabolic response in live nerves was achieved by segmentation of ex vivo injured sciatic nerves (tibial and common peroneal branches) into five equal length segments beginning at the distal nerve stump (S1) (site of injury) and extending proximally (S5), with comparison to equal length segments from naive uninjured nerves (Fig. 5B). Degenerating nerve tissue distal to the site of transection was not included in this analysis. Nerve segments closer to the site of nerve injury (S1 and S2) exhibited elevated basal respiration, as measured by real-time cellular metabolic flux analysis (Seahorse bioanalyzer) (Fig. 5C). The extracellular acidification rate (ECAR), which measures acidification primarily by lactate generated from glycolysis with some tricarboxylic acid (TCA) cycle contributions, was also significantly elevated in nerve segments closest to the site of injury (S1 and S2), relative to naive nerves (Fig. 5D). Key parameters of mitochondrial respiration were pharmacologically dissected using mitochondrial complex-specific inhibitors, revealing that the rate of oxygen consumption attributable to excessive mitochondrial proton leak as well as maximum respiration were significantly elevated in nerve segments closest to the site of injury (S1 and S2) compared to naive nerves (Fig. 5E and fig. S5A). This was accompanied by a marked decrease in spare respiratory capacity in segments closest to the injury site (S1; Fig. 5F). In accordance with these indicators of metabolic dysfunction, the efficiency of ATP generation, as measured by the fraction of respiration rate coupled to ATP production, declined in the injured nerve segments closest to the injury site (normalized ATP production–coupled respiration; Fig. 5G). The normalized ATP-coupled respiration, plotted as OCR (% of total), is used to prevent condition differences in total OCR from masking changes in respiratory efficiency. Although overall ATP production was modestly increased (fig. S5B), the decline in efficiency of ATP production and highly elevated mitochondrial proton leak with decreased spare respiratory capacity are consistent with impaired mitochondrial function, and together with concomitantly elevated ECAR support a metabolic shift toward glycolysis in the injured nerve (Fig. 5). Collectively, these findings reveal altered metabolism and parameters of mitochondrial function in sciatic nerves at day 7 following nerve injury and are concordant with metabolic disturbances reported at this time frame in other settings of peripheral nerve injury (*35*).

**Fig. 5.**
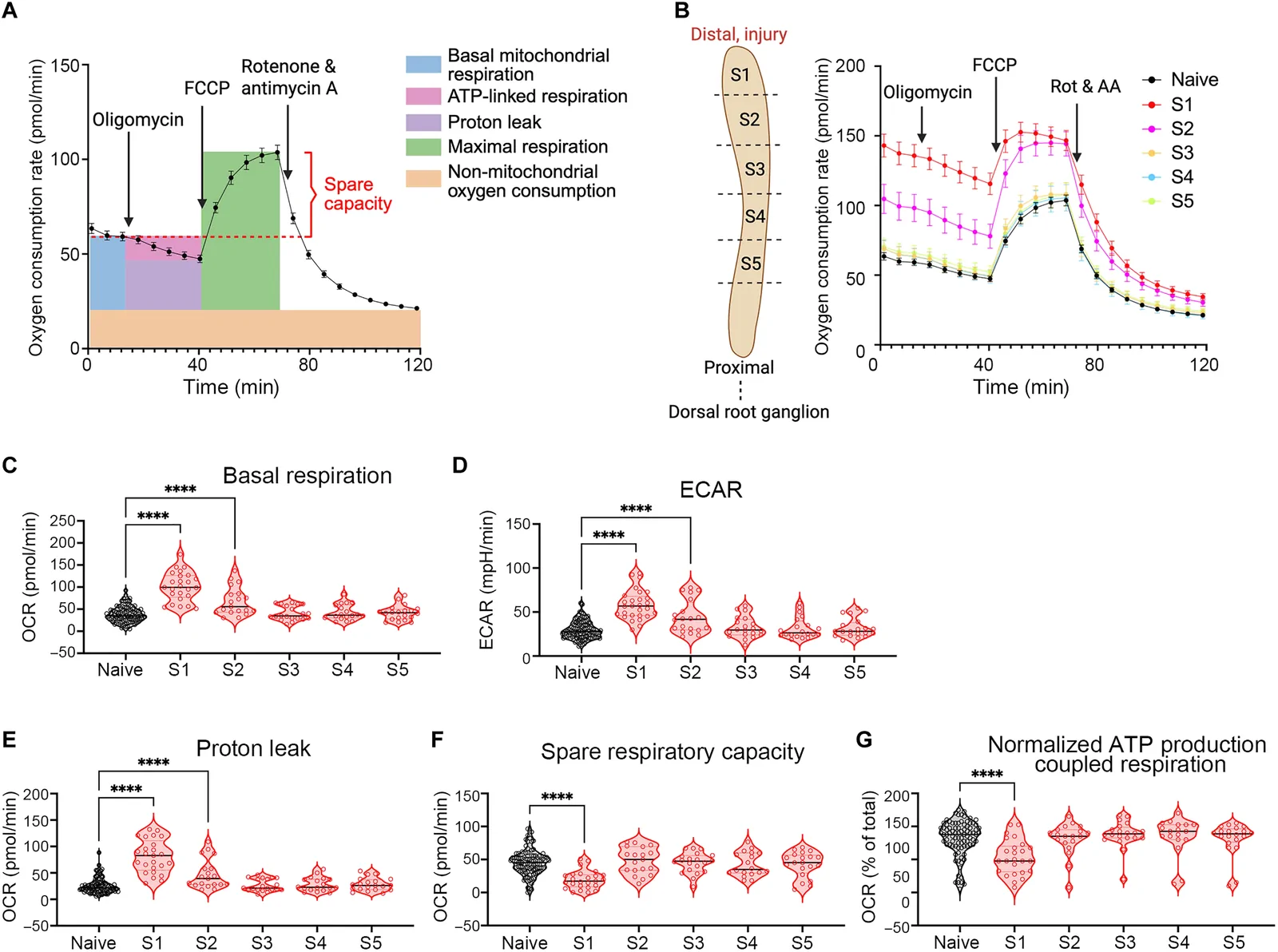
Nerve injury induces metabolic dysregulation in axons of injured nerve. (**A**) Schematic of the Seahorse Cell Mito Stress Test assay showing calculable respiratory parameters. The Seahorse analyzer measures the OCR of sciatic nerve segments before and after the sequential addition of oligomycin (ATP synthase inhibitor), FCCP (collapses proton gradient and disrupts mitochondrial membrane potential), rotenone (Rot; complex I inhibitor), and antimycin A (AA; complex III inhibitor). (**B**) OCR of naive and injured sciatic nerve segments at 7 days postinjury (SNI) in the Seahorse Cell Mito Stress Test assay. Sciatic nerves were isolated and cut into 1.5-mm-long segments; injured nerve segments were labeled as S1 to S5, with S1 representing the nerve stump of the injury site. Each segment was placed into a single well on a 96-well spheroid microplate. *N* = 18 to 20 male mice per condition. (**C** to **G**) Quantification for respiratory parameters of basal respiration (C), basal ECAR (D), proton leak (E), spare respiratory capacity (F), and normalized ATP production–coupled respiration [(G); normalized for basal respiration] in naive and injured sciatic nerve segments (S1 to S5) at 7 days postinjury. One-way ANOVA; Dunnett’s multiple comparisons test; *N* = 18 to 20 male mice per condition. ****P < 0.0001.

Considering the lowered let-7 miRNA levels in injured sciatic nerves and the established roles of let-7 miRNAs in governing growth and metabolism, we tested the metabolic impact of restoring injury-decreased let-7 miRNA. Ex vivo day 7 post-SNI sciatic nerves were divided into five equal length segments from the distal nerve stump (S1) to the most proximal segment (S5) (furthest from injury site). All segments were immediately maintained in a humidified oxygenated chamber and incubated 1.5 hours with either vehicle (untreated), a nontarget miRNA mimic (nontarget), or let-7 miRNA mimics at 4 or 8 pmol. Treatment of naive or proximal injured nerve segments with nontarget and let-7 miRNA mimics had negligible effects on metabolic responses, consistent with the minimal impacts of nerve injury (fig. S6, A to E). Given that our data (Fig. 5) showed the most pronounced metabolic alterations to be in distal nerve segments closest to the site of injury, we anticipated that any effect of let-7 miRNA treatment on metabolic flux analysis might be more evident in the S1 nerve segment. Whereas control nontarget miRNA mimics exhibited no effect on respiratory function measurements in injured nerve segments (Fig. 6, A and D to H, and fig. S6, F and G), let-7 miRNA mimics produced a dose-dependent shift in the oxygen consumption of the injured nerve (S1, segments closest to injury site) (Fig. 6B) back toward the parameters observed in uninjured (fig. S6A) or in more proximal (S3 to S5) nerve segments (Fig. 6C, S3 to S5 averaged raw OCR measurements; fig. S6, C to E, individual segments). Pharmacological dissection of respiratory parameters in the S1 injured segments using mitochondrial complex-specific inhibitors further demonstrated that let-7 miRNA mimics significantly rescued basal OCR and reduced injury-induced excessive mitochondrial proton leak respiration toward levels in uninjured nerve (Fig. 6, D and E). We did not detect a significant impact of let-7 miRNA mimics under our treatment conditions on either the injury-induced elevation in acidification (ECAR, arising from glycolysis with TCA cycle contributions) or on spare respiratory capacity (Fig. 6, F and G) of injured sciatic nerves; a caveat is that these effects could be more difficult to detect because the primary injury-induced elevations are more subtle than for the injury-induced changes in basal respiration and proton leakage. Notably, the injury-induced decline in overall efficiency of ATP generation (normalized ATP production–coupled respiration) was also rescued toward uninjured levels by treatment with let-7 miRNA mimics (Fig. 6H). Together, these findings implied that locally replenishing the injury-induced loss of let-7 miRNAs with let-7 miRNA mimics in the sciatic nerve was sufficient to significantly improve metabolic disturbances following nerve injury.

**Fig. 6.**
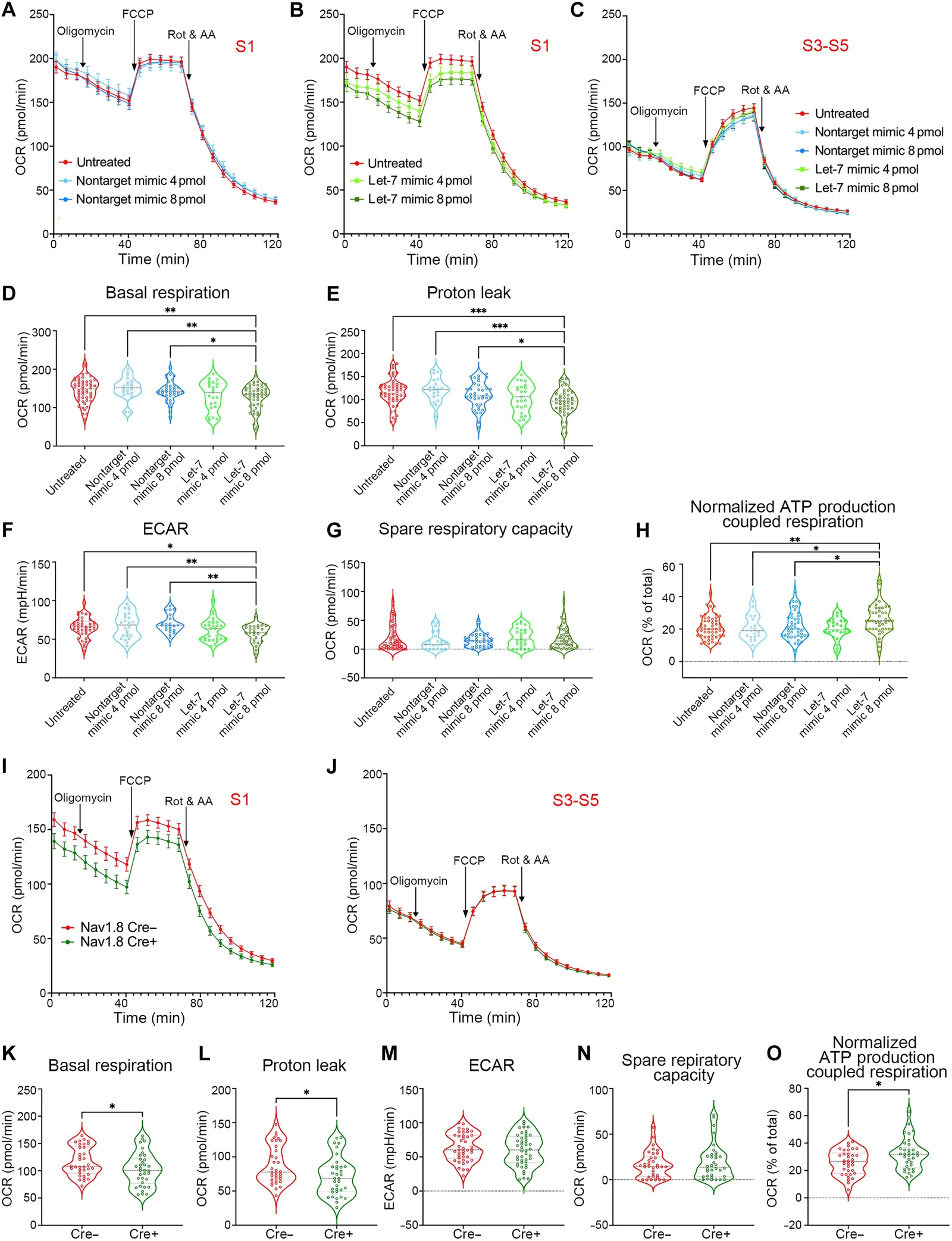
Lin28/let-7 miRNA pathway mediates metabolic dysregulation following nerve injury. (**A** and **B**) OCR of injured S1 at 7 days postinjury (SNI), untreated or with 4 and 8 pmol of nontarget (A) or let-7 miRNA mimic (B) in the Seahorse Cell Mito Stress Test assay of nerve segments. *N* = 12 to 25 male mice per condition. (**C**) Averaged OCR of injured S3 to S5 nerve segments at 7 days postinjury (SNI) untreated or with 4 and 8 pmol of nontarget or let-7 mimic. *N* = 12 to 25 male mice per condition. (**D** to **H**) Calculated basal respiration (D), proton leak (E), basal ECAR (F), spare respiratory capacity (G), and normalized ATP production–coupled respiration (H; normalized to basal respiration) in injured S1, 7 days postinjury, untreated or with 4 and 8 pmol of nontarget or let-7 mimic One-way ANOVA; Fisher’s least significant difference (LSD) multiple comparisons test; *N* = 12 to 25 male mice per condition. (**I**) OCR of Lin28a^flox/−^/Nav1.8^Cre−^ and Lin28a^flox/−^/Nav1.8^Cre+^ injured S1 nerve at 7 days postinjury (SNI) in the Seahorse Cell Mito Stress Test assay. *N* = 20 of both sexes in Lin28a^flox/−^/Nav1.8^Cre−^; *N* = 19 of both sexes in Lin28a^flox/−^/Nav1.8^Cre+^. (**J**) Averaged OCR of Lin28a^flox/−^/Nav1.8^Cre−^ and Lin28a^flox/−^/Nav1.8^Cre+^ injured S3 to S5 nerve segments at 7 days postinjury (SNI). *N* = 20 mice of both sexes in Lin28a^flox/−^/Nav1.8^Cre−^; *N* = 19 mice of both sexes in Lin28a^flox/−^/Nav1.8^Cre+^. (**K** to **O**) Calculated basal respiration (K), proton leak (L), basal ECAR (M), spare respiratory capacity (N), and normalized ATP production–coupled respiration (O; normalized to basal respiration) in Lin28a^flox/−^/Nav1.8^Cre−^ and Lin28a^flox/−^/Nav1.8^Cre+^ injured S1 at 7 days postinjury. Two-tailed unpaired Student’s *t* test with Welch’s correction; *N* = 20 mice of both sexes in Lin28a^flox/−^/Nav1.8^Cre−^; *N* = 19 mice of both sexes in Lin28a^flox/−^/Nav1.8^Cre+^. *P < 0.05, **P < 0.01, and ***P < 0.001.

### Sensory neuron Lin28 governs metabolic responses following nerve injury

To evaluate the specific role of the Lin28/let-7 pathway in sensory neurons with axons in the sciatic nerve, we next probed metabolic responses to injury using a genetic approach. Selective Lin28 knockout in Nav1.8 lineage sensory neurons, predominantly nociceptors, was achieved by generating C57BL/6J-backcrossed conditional Lin28a^flox/−^ x Nav1.8 Cre recombinase (Lin28a^flox/−^/Nav1.8^Cre+^) mice. Lin28a^flox/−^ mice, negative for Nav1.8^Cre^, (Lin28a^flox/−^/Nav1.8^Cre−^) served as controls. Loss of Lin28a was confirmed by bulk RNA-seq of sciatic nerves from Lin28a^flox/−^/Nav1.8^Cre−^ and Lin28a^flox/−^/Nav1.8^Cre+^ mice (fig. S6H). Metabolic flux analyses carried out on ex vivo sciatic nerves at day 7 post-SNI recapitulated the expected injury-induced elevation in basal respiration near the injury site in control Lin28a^flox/−^/Nav1.8^Cre−^ mice (Fig. 6I), which was comparable with the response in wild-type (WT) C57BL/6J mice (Fig. 5). However, selective loss of Lin28a from sensory neurons (Lin28a^flox/−^/Nav1.8^Cre+^) lowered the high oxygen consumption parameters of the injury-adjacent nerve segments (S1) (Fig. 6I), shifting metabolism toward the indices of more proximal (S3 to S5), or contralateral uninjured, nerve segments (Fig. 6J, S3 to S5 averaged raw OCR measurements; fig. S6, I to L, individual segments). Consistent with an inability to up-regulate Lin28a in response to injury and consequent failure to down-regulate let-7 miRNAs, the metabolic response to nerve injury in Lin28a^flox/−^/Nav1.8^Cre+^ sciatic nerve resembled the effect of locally elevating let-7 miRNAs with miRNA mimics (Fig. 6, K to O), including a rescue of the overall efficiency of ATP generation toward uninjured levels (Fig. 6O). Unlike other metabolic parameters, injury-induced ECAR elevation was reduced by let-7 mimics but did not appear significantly affected by the loss of Lin28a from sensory neurons (Lin28a^flox/−^/Nav1.8^Cre+^) (Fig. 6, F and M); this could reflect differences in cell selectivity or biological effects of Lin28 relative to let-7 miRNAs.

### Loss of Lin28 suppresses postinjury neuronal hyperexcitability

Disturbances in metabolic and mitochondrial function are closely associated with the development of pathological neuronal activity and hyperexcitability in both the central and peripheral nervous systems (*40*, *42*, *78*–*80*). In addition to long-term mitochondrial disorders, neuronal hyperexcitability can result from acute mitochondrial dysfunction, such as in response to chemical agents which dissipate the mitochondrial proton gradient to uncouple the electron transport chain from ATP synthesis (*81*). In the context of nerve injury, injured axons can also signal to nearby uninjured “spared” axons (by incompletely defined mechanisms) to promote nerve injury–associated plasticity and changes in the activity of those uninjured neurons (*82*–*86*). Given our data revealing metabolic dysfunction, including increased mitochondrial proton leak downstream of lowered let-7 miRNA levels in nerve injury, we next sought to evaluate potential functional impacts on sensory neuron activity in both injured and spared peripheral sensory neurons.

We began to evaluate potential differences in neuronal physiology of nociceptive DRG neurons from mice expressing Lin28a (Lin28a^flox/−^/Nav1.8^Cre−^) or deficient in Lin28a (Lin28a^flox/−^/Nav1.8^Cre+^) by conducting Fura-2 ratiometric calcium imaging on cultured dissociated DRG neurons from these mice. To enrich the analyses for neurons likely expressing NaV1.8, we confined our assessments to small diameter neurons, less than 1250 μm^2^. We observed a slightly reduced average relative basal calcium level in presumptively Lin28-deficient neurons, compared to controls (Fig. 7A and fig. S7A). To explore the excitability of these neurons in vitro, we stimulated them in sequence using submaximal KCl depolarization (15 mM) and submaximal capsaicin (10 nM), with an intervening washout. Analyzed calcium traces showed a trend, which did not reach statistical significance, toward a reduced proportion of cells responsive to KCl depolarization in the Lin28-deficient neurons compared to controls. Comparison of the amplitude of calcium response to KCl depolarization among the top responding cells revealed a similar trend toward reduced responsiveness in the Lin28-deficient neurons that did not reach statistical significance when analyzed on a “per mouse” basis but did show statistical significance when compared across individual analyzed coverslips (Fig. 7, B and C, and fig. S7, A and B). By comparison, for capsaicin, there were no differences between genotypes in either the proportion of responsive cells or the mean amplitudes among top responders (Fig. 7, D and E, and fig. S7, A and C). Collectively, these analyses did not show notable differences but suggested that Lin28 deficiency might result in modest alterations of excitability and/or calcium handling in naïve DRG neurons in an in vitro setting. Further investigation of potential neurophysiological impacts of Lin28 deficiency necessitated evaluation in the setting of in vivo nerve injury.

**Fig. 7.**
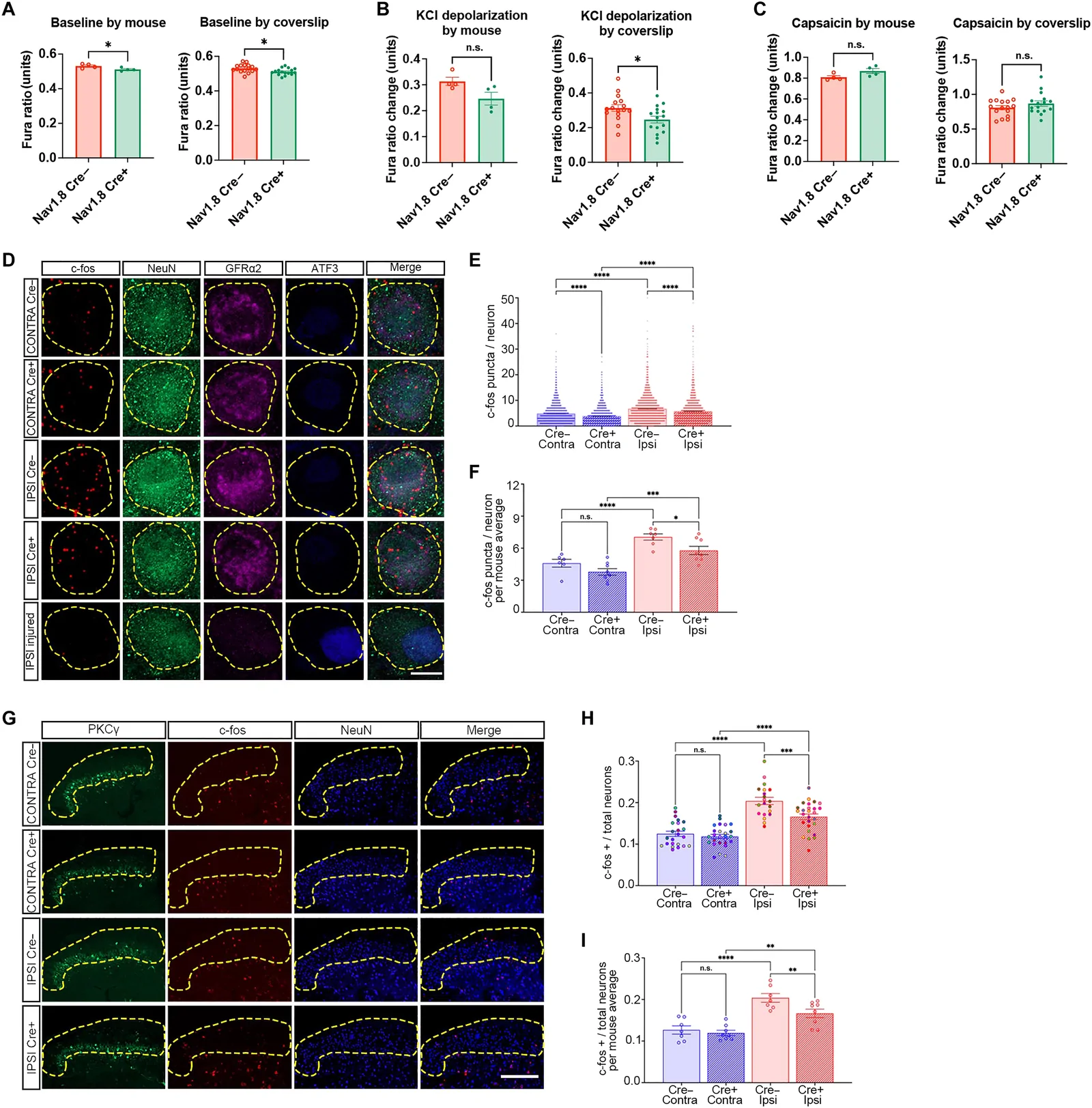
Lin28 deficiency reduces neuronal activity elevation post–nerve injury. (**A** to **C**) Fura-2 ratiometric calcium imaging of DRG neurons cultured from Lin28a^flox/−^/Nav1.8^Cre−^ and Lin28a^flox/−^/Nav1.8^Cre+^ mice. (A) Baseline relative calcium quantified by mouse (left) or by coverslip (right). (B) Mean 15 mM KCl-evoked ratio change in top responders (top 9% of cells) per coverslip, quantified by mouse (left) or by coverslip (right). (**C**) Mean capsaicin-evoked change in top responders (top 40%, based on cross-coverslip responses) by mouse (left) or coverslip (right). Symbols represent individual mice (*n* = 4 per genotype) or coverslips (*n* = 4 per mouse). *P* values from two-tailed, unpaired Welch’s *t* test. (**D**) Representative c-fos mRNA RNAscope confocal projections in contralateral and ipsilateral uninjured and injured neurons from Lin28a^flox/−^/Nav1.8^Cre−^ and Lin28a^flox/−^/Nav1.8^Cre+^ DRG, 7 days postinjury (SNI); soma (outlined), neurons (NeuN), IB4-binding nociceptive neurons (GFRα2), and injured neurons (ATF3). Scale bar, 10 μm. (**E** and **F**) c-fos mRNA puncta per cell (E) and average per mouse (F), from contralateral and ipsilateral uninjured GFRα2-positive neurons in Lin28a^flox/−^/Nav1.8^Cre−^ and Lin28a^flox/−^/Nav1.8^Cre+^ DRG, 7 days postinjury (SNI). *N* = 2000 to 3000 cells from both sexes in Lin28a^flox/−^/Nav1.8^Cre−^ (seven mice); *N* = 2000 to 3000 cells in Lin28a^flox/−^/Nav1.8^Cre+^ (eight mice). (**G**) Representative c-fos mRNA RNAscope in spinal cord from Lin28a^flox/−^/Nav1.8^Cre−^ and Lin28a^flox/−^/Nav1.8^Cre+^ mice at 7 days postinjury (SNI). PKCγ marks lamina II edge; laminae I and II are outlined. Scale bar, 200 μm. (**H** and **I**) c-fos–positive/total neurons in laminae I and II (H) and averaged per mouse (I) in contralateral and ipsilateral spinal cord from Lin28a^flox/−^/Nav1.8^Cre−^ and Lin28a^flox/−^/Nav1.8^Cre+^ mice, 7 days postinjury (SNI). The same mouse is color coded consistently (H). *N* = 30 to 40 spinal cord sections from both sexes in Lin28a^flox/−^/Nav1.8^Cre−^ (seven mice) or Lin28a^flox/−^/Nav1.8^Cre+^ (eight mice). [(E), (F), (H), (I)] One-way ANOVA; Fisher’s LSD multiple comparisons. Error bars represent the SEM; *P < 0.05, **P < 0.01, ***P < 0.001, and ****P < 0.0001.

Using induction of immediate early gene c-fos transcript levels as an established sensitive readout of sensory neuron activity (*87*–*90*), we evaluated c-fos expression by single-molecule in situ hybridization in DRG neurons at 7 days postinjury (SNI). Immunostaining for ATF3 was used to differentiate injured and uninjured sensory neurons, whereas costaining for GFRα2, which is expressed by isolectin B4 (IB4)–binding nonpeptidergic nociceptive sensory neurons, was used to refine analysis to a homogeneous subset of nociceptive sensory neurons in L3/L4 DRG (Fig. 7D and fig. S7D). Uninjured, spared, nociceptive DRG neurons in mice either expressing Lin28a (Lin28a^flox/−^/Nav1.8^Cre−^) or deficient in Lin28a (Lin28a^flox/−^/Nav1.8^Cre+^) both exhibited elevated c-fos ipsilateral to the SNI compared to contralateral, as anticipated (Fig. 7, D and E). However, the c-fos induction following SNI was significantly lower in uninjured nociceptive neurons ipsilateral to injury in Lin28a-deficient mice compared to in control Cre-negative mice, both at the individual cell level and when evaluated by overall mouse averages (Fig. 7, E and F). L3/L4 DRG quantifications are presented averaged (Fig. 7E) and individually (fig. S7E). Although contralateral Cre-positive DRG nociceptive neurons exhibited lower c-fos expression compared to contralateral Cre-negative nociceptive DRG neurons when evaluated at the individual cell level (Fig. 7, D and E), this difference lacked statistical significance when data were compiled on a “per mouse” average basis (Fig. 7F and fig. S7E). Expression of an additional immediate early gene, Zif268, exhibited similar trends (fig. S7, F and G), although a higher variability of induction from baseline expression (c-fos SEM: 0.29; Zif268 SEM: 0.68), as has been reported by others (*91*), precluded its effective use without large numbers of mice. ATF3-positive (injured) neurons showed negligible c-fos (most puncta counts were zero) at this 7 days postinjury time point, as well as becoming GFRα2 negative, and were not further analyzed (*92*).

To evaluate whether the lower activity, indicated by c-fos induction, in Lin28a-deficient peripheral sensory neurons might lead to decreased ascending sensory transmission in the central nervous system, we examined c-fos expression in the sensory neurons within the spinal cord dorsal horn, which receive and process nociceptive signals transmitted by primary afferent neurons. Nociceptive primary afferents terminate in the superficial laminae (I and II) of the dorsal spinal cord; protein kinase C γ (PKCγ) immunostaining was used to mark the bottom of lamina II (*93*–*95*) with costaining for NeuN to permit evaluation of c-fos expression in second-order nociceptive neurons (Fig. 7G). c-fos was evaluated at 7 days postinjury, a time point when Lin28 induction and lowered let-7 miRNA levels were most pronounced in the injured sciatic nerve (Fig. 2F). Quantification of c-fos, in the absence of additional peripheral stimulation, indicated that nerve injury elevated the activity of second (or higher)–order nociceptive neurons in the spinal cord ipsilateral to the injury. This elevation was observed in both Cre-positive mice (Lin28a^flox/−^/Nav1.8^Cre+^) and Cre-negative mice (Lin28a^flox/−^/Nav1.8^Cre−^), as measured by the total c-fos–positive spinal cord sensory neuron number and when evaluated by the average number of c-fos–positive spinal cord neurons per mouse (Fig. 7, G and H). However, the injury-induced c-fos induction in spinal cord sensory neurons was significantly lower in mice with Lin28a-deficient primary nociceptive sensory neurons compared to control mice expressing Lin28a in primary nociceptive neurons (Fig. 7, H and I). In agreement with our observations in DRG sensory neurons (Fig. 7, D to F), Lin28a deficiency in primary nociceptive neurons did not produce a detectable effect on c-fos activity in spinal cord nociceptive neurons in the control setting contralateral to SNI (Fig. 7, H and I). Expression of an additional immediate early gene marker of neuronal activity, Zif268, exhibited trends similar to c-fos, although again showed higher variability in induction (fig. S7, H and I). As expected, significant elevations in nociceptive neuron activity in the peripheral and central nervous systems were observed only in the ipsilateral context of nerve injury and not in the contralateral control context. Together, our data show that mice with Lin28a-deficient primary nociceptive neurons exhibit significantly less c-fos induction in ipsilateral uninjured nociceptive neurons of the DRG and in ipsilateral spinal cord dorsal horn following nerve injury, indicating reduced injury-induced elevation of nociceptive activity compared to control mice. This ipsilateral impact of Lin28a deficiency, without a significant contralateral effect, is consistent with importance of the injury-induced Lin28 up-regulation in ipsilateral nerves (Fig. 1) in producing both altered metabolism in injured nociceptive neurons and the concurrent elevated activity of nearby uninjured nociceptive neurons (Figs. 7, D to I, and 8).

**Fig. 8.**
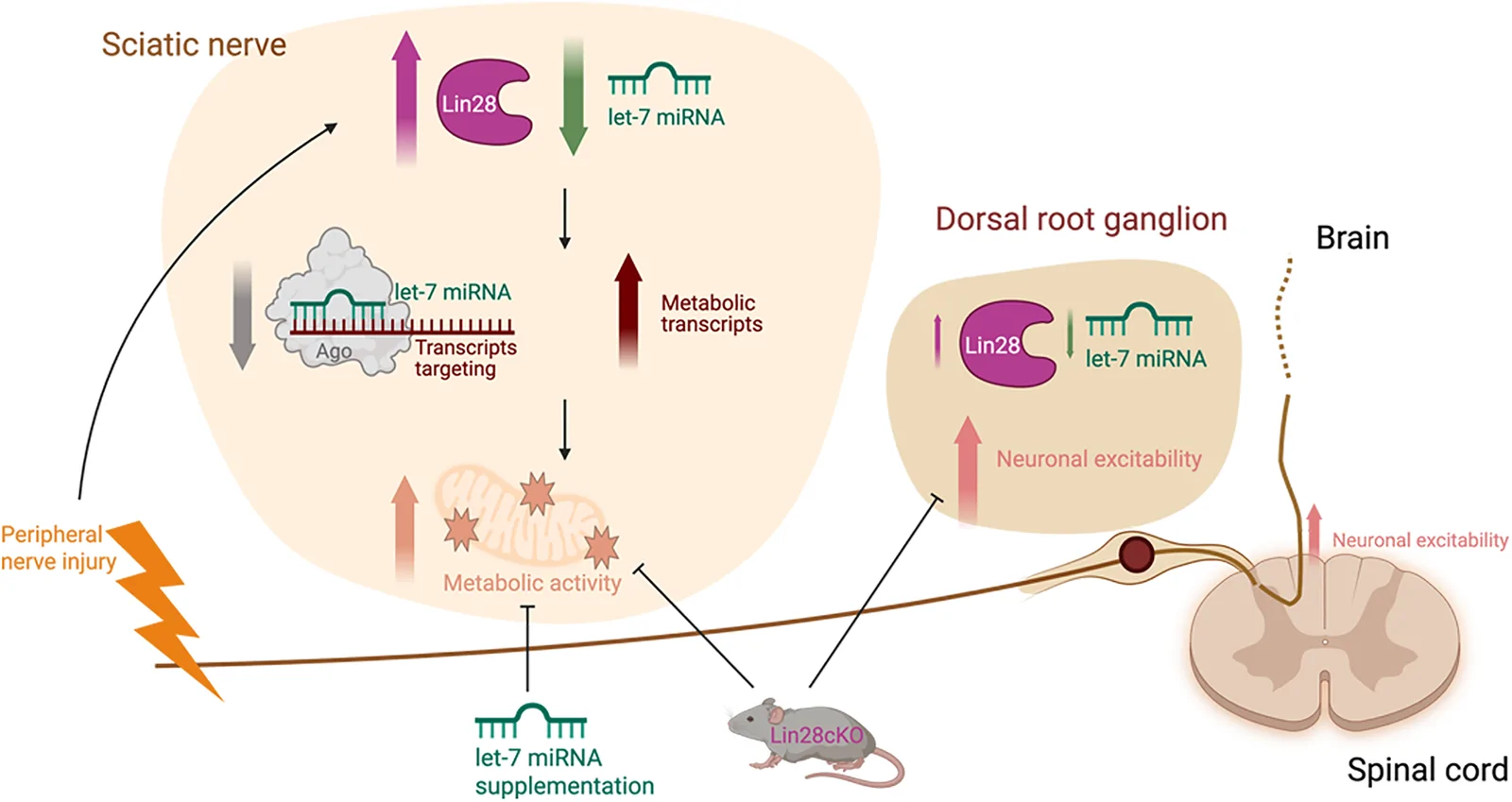
Summary of results. Nerve injury induced Lin28 RNA binding proteins and lowered levels of let-7 miRNAs, which are negatively regulated by Lin28. A metabolic response of altered cellular respiration occurred following peripheral nerve injury. Genome-wide profiling of miRNA:target RNA interactions in Nav1.8^+^ sensory neurons revealed that nerve injury led to derepression of gene targets by let-7 miRNAs, including targets involved in mitochondrial respiration and glycolysis. Replenishment of the lowered let-7 miRNAs in injured sciatic nerves inhibited the metabolic response with mitigation of the injury-induced increases in mitochondrial proton leak and basal respiration and enhancement of ATP production–coupled respiration. Consistently, selective knockout of Lin28a in peripheral Nav1.8 lineage sensory neurons also mitigated injury-induced elevation of mitochondrial proton leak and basal respiration, as well as inhibiting the heightened activity of nociceptive neurons in response to nerve injury. Created in BioRender. Witwer, K. (2026) https://BioRender.com/72lyy6x.

## DISCUSSION

Peripheral nerve injury results in extensive structural and functional reorganization that involves both lesioned and unlesioned axons and extends from the peripheral to the central nervous system. The capacity of nerve injury to produce prolonged elevated activity in primary peripheral, as well as central, nociceptive, neurons is a critical biological outcome resulting from multiple types of peripheral trauma. The complex metabolic response to nerve injury is also well documented, with elevated neuronal activity and modified activity set points shown to arise in response to altered metabolic function (*37*, *40*, *78*, *80*, *81*, *96*–*99*). However, gaps have remained in understanding the molecular pathways responding to injury to affect metabolism as well as neuronal activity. We define a mechanism coupling induction of the conserved Lin28/let-7 pathway, a key driver in mammalian metabolism (*22*, *32*, *100*), to altered metabolic respiration in injured sensory neurons and their peripheral axons, as well as to injury-heightened activity in sensory neurons of the peripheral and central nervous systems (Fig. 8).

Let-7 miRNAs have evolutionarily conserved roles repressing pro-growth mRNAs to exert regulation of many growth-related processes including metabolic reprogramming, cell survival, development, and oncogenesis (*15*, *21*, *101*, *102*). Apart from development, let-7 miRNAs also play prominent roles in metabolic homeostasis, particularly in glucose and insulin signaling, which include the regulation of targets such as AMP-activated protein kinase (AMPK) in the contexts of obesity and mitochondrial biology (*103*, *104*). In this study, cell-type–selective genome-wide profiling of miRNA:target RNA interactions revealed that nerve injury led to derepression of gene targets by let-7 miRNAs in Nav1.8 lineage sensory neurons and afferents from injured sciatic nerves. Nerve injury induced both Lin28a and Lin28b RNA from low basal levels in nociceptive sensory neurons and produced a concomitant decline in let-7 miRNAs, which are negatively regulated by Lin28 protein. Profiling highlighted newly identified let-7 metabolic targets, including mitochondrial transcripts, as well as validating previously identified metabolic targets, such as genes for the insulin receptor (INSR), pyruvate dehydrogenase kinase 2 (PDK2), and c-Myc, which are known to be involved in enhancing glycolysis and which were derepressed in the ipsilateral nerve with reduced miRNA targeting and elevated transcript levels upon injury. Impaired peripheral nerve INSR signaling has been previously linked to mechanical hyperalgesia and thermal hypoalgesia in the context of diabetes (*105*), and up-regulation of INSR signaling is reported to enhance axon health and regeneration following nerve injury (*106*–*108*). PDK2 inhibits pyruvate dehydrogenase to promote glycolysis and lactic acid production, at the expense of OXPHOS. PDK2 up-regulation in the DRG following nerve injury can promote lactic acid surge and pain, whereas ablation of PDK2 (and PDK4) has been shown to protect mice against sciatic nerve damage and attenuate some forms of hyperalgesia (*109*–*111*). The c-Myc transcription factor, a master regulator of growth and metabolism, has been reported to be elevated in sensory neurons and Schwann cells, following peripheral nerve injury. C-Myc can promote expression of glycolytic as well as repair-associated genes in the context of preconditioning peripheral nerve injury, and its expression has been shown to enhance sensory axon numbers at injury borders (*112*, *113*). In our work, injury-induced Lin28 elevation and decreased let-7 miRNA levels would be expected to allow up-regulation of the let-7 targets, INSR, PDK2, and c-Myc.

Additionally, we found that the Lin28/let-7 pathway contributed to injury-induced metabolic disturbances on a functional level. Locally supplementing the injury-associated loss of let-7 miRNAs in sciatic nerves, or inhibiting Lin28 induction by injury with the Nav1.8+ sensory neuron-selective Lin28a knockout, each significantly alleviated injury-associated metabolic disruptions to support a previously unrecognized role for the Lin28/let-7 axis in regulating metabolic homeostasis in peripheral sensory neurons post–nerve injury. Elevated oxygen consumption and a decline in efficiency of ATP generation in injured nerves could be rescued, at least in part, by replenishing let-7 miRNA levels or by selective sensory neuron knockout of Lin28a. Unlike other metabolic parameters, the injury-induced elevation in ECAR, predominantly due to glycolysis with some TCA cycle contribution (based on rotenone profile), responded differentially to loss of Lin28 compared to let-7 replenishment. Let-7 miRNA mimics reduced the injury-induced ECAR elevation, whereas loss of Lin28 did not; reasons for this remain to be explored but could reflect differences such as the cell type selectivity of the genetic approach taken for Lin28. Recent evidence outside the context of nerve injury suggests that sensory neurons expressing Nav1.8 also participate in metabolic control and energy balance at the organismal level (*114*).

It is possible that double knockout of Lin28a and Lin28b in nociceptive neurons might further blunt both metabolic and elevated activity response to nerve injury because both Lin28 paralogs were induced by injury; however, prolonged loss of both Lin28 paralogs could also risk impacts to cell health (*11*, *102*, *115*, *116*). Although our results indicated that a gain in let-7 miRNA levels or a loss in Lin28 produced congruent effects on metabolism and neuronal activity, we do not rule out that Lin28 could have additional let-7 independent effects that are masked in our system. Whereas Lin28a/b RNA binding proteins potently inhibit let-7 family miRNA biogenesis (*117*), Lin28 proteins also bind RNAs other than let-7 (*118*–*120*), although some evidence indicates that non-miRNA binding predominantly functions to sequester Lin28 away from miRNA binding rather than as an alternative physiological gene regulatory mechanism (*121*). Defects in mitochondrial metabolism have been linked to neuronal hyperexcitability (*37*, *40*, *80*, *96*, *97*, *122*) and metabolic genes are prominent let-7 miRNA targets, but additional let-7–targeted transcripts in sensory neurons may also undergo altered targeting following injury that contributes to elevated neuronal activity or other downstream responses. Recently, it was reported that inhibition of the mitochondrial ETC was protective against capsaicin-evoked death and that lower basal expression of ETC components in TRPV+ nociceptive sensory neurons could contribute to their resilience to excitotoxic damage (*123*). Given these findings, cytoprotection might be speculated to be an additional outcome coupled to the lowered mitochondrial function in injured sciatic nerves. Mildly increased mitochondrial proton leak, while lowering efficiency of ATP production, can, in some instances, be cytoprotective by lowering production of reactive oxygen species (ROS) (*124*). There is evidence that these potential benefits are outweighed in the longer term by the detrimental energy-depleting effects of excessive mitochondrial proton leak, both in reports from cardiac tissue, in which limiting leak restores cellular function (*125*), and in peripheral nerves where adoptive transfer of healthy mitochondria reduces axonal degeneration and neuropathic pain (*126*).

Our results are consistent with prior studies reporting Lin28 up-regulation in DRG lysates using a nerve crush and regeneration model (*127*, *128*), as well as Lin28 induction in other injury settings including axolotl forelimb amputation, hepatocyte alcohol injury, cardiomyocyte hypoxia, acute lung injury, and obesity-induced Lin28 in the hypothalamus (*20*, *129*–*132*). Notably, single-molecule studies allowed us to map Lin28 induction to injured sensory neurons and to make the unanticipated observation of a more pronounced and sustained Lin28 up-regulation in peripheral axons relative to sensory neuron somas. Our results add to existing evidence supporting the importance of local signaling and RNA regulation within nerves and axons in mediating functional plasticity following peripheral nerve injury (*133*–*136*). This is further supported by critical roles for multiple RNA binding proteins in the generation of pain following peripheral nerve injury, and their proposed targeting for pain therapeutics (*137*, *138*). The signaling pathway responsible for Lin28 induction following nerve injury remains to be determined; however, our lab and others have previously demonstrated that MAPK/ERK-dependent Lin28 induction (*11*–*14*) and prolonged MAPK/ERK activation are a well-characterized response to nerve injury (*139*–*142*).

Pivotal roles for the Lin28/let-7 pathway in regulating energy homeostasis and cellular metabolic reprogramming have been reported in diverse biological contexts (*22*, *32*, *100*). In mammals, Lin28 and let-7 miRNAs are known to prominently regulate glucose metabolism: Elevated Lin28 or lowered let-7 miRNA levels can enhance glycolytic metabolism, including derepressing let-7 targets in the insulin-PI3K-mTOR pathway (*30*) and improving insulin sensitivity to glucose (*29*, *30*). In the setting of cancer, elevated Lin28 has been linked to the metabolic Warburg effect and cancer progression with lowered let-7 miRNA levels promoting glycolysis at the expense of mitochondrial OXPHOS through let-7 targets including PDK1 (*143*) and hexokinase 2 (HK2) (*144*). Lowered let-7 miRNA levels have been previously reported to impair mitochondrial respiration, including increasing proton leak, in cultured mouse skeletal muscle cells (*145*) and a myoblast cell line (*146*). In agreement with a prior characterization of altered bioenergetic function following sciatic nerve injury by ligation (*35*), we observed an increase in mitochondrial proton leak accompanied by elevated basal mitochondrial respiration and reduced ATP production–coupled and spare respiration at day 7 post–nerve injury in our study. These injury-induced metabolic alterations were significantly affected by either preventing Lin28a induction in sensory neurons or by using let-7 miRNA mimics to inhibit reduction in let-7 miRNAs. To our knowledge, this critical link between regulation of the Lin28/let-7 miRNA pathway and the metabolic response to nerve injury has not been previously reported in the mammalian nervous system. In future studies, it will be informative to investigate how Lin28/let-7–mediated metabolic changes in injured neurons and parallel changes in the activity of spared uninjured sensory neurons may be linked or, alternatively, whether these represent cell autonomous roles for the Lin28/let-7 pathway in responding to nerve injury. It will also be important to test whether the Lin28/let-7 miRNA pathway may more broadly participate in the metabolic regulation and elevated sensory neuronal activity observed in alternative settings of nerve injury such as in diabetes or chemotherapy-induced peripheral neuropathies (*147*–*150*). Collectively, our findings increase mechanistic understanding of the signaling pathways mediating elevated neuronal activity and regulation of metabolism and mitochondrial biology in sensory neurons responding to nerve injury.

## MATERIALS AND METHODS

### Experimental model details

Nav1.8-Cre [B6.129(Cg)-Scn10atm2(cre)Jwo/TjpJ; stock #036564, RRID: IMSR_JAX:036564], R26-LSL-tAgo2 [B6.Cg-Gt(ROSA)26Sortm1(CAG-GFP/Eif2c2)Zjh/J; stock #017626, RRID: IMSR_JAX:017626], and WT mice (stock #000664, RRID:IMSR_JAX:000664) on the C57BL/6J background were obtained directly or indirectly from the Jackson Laboratory. The mouse line with conditional tAgo2 expression in nociceptive neurons was generated by crossing B6.Nav1.8-Cre (*67*) and R26-LSL-tAgo2 (*66*). The mouse line with conditional Lin28a knockout in nociceptive neurons was generated by crossing B6.Nav1.8-Cre (*67*) and Lin28^fl/−^ (*30*). All experiments were performed with age- and sex-matched mice from the same or parallel mating cages, unless otherwise specified. Biochemical experiments (in situ hybridization, immunostaining, digital PCR, Seahorse metabolic assay, and chimeric RNA-seq) were performed on male mice unless otherwise stated. Sequencing experiments were conducted in both sexes.

Adult mice were group housed (up to five sex-matched animals per cage) on a standard 14-hour light/10-hour dark cycle at 24°C and received water and food ad libitum. All procedures related to the care and treatment of animals were approved by the Johns Hopkins School of Medicine Institutional Animal Care and Use Committee (IACUC, protocol MO25M404) and were in compliance with the Association for Assessment of Laboratory Animal Care (AAALAC) guidelines for animal use. All mice in this study were maintained on the C57BL/6J background.

### Method details

#### Genotyping PCR

Genomic DNA was extracted from mouse tails or ears using alkaline lysis solution (25 mM NaOH and 0.2 mM EDTA) and neutralized with 40 mM tris-HCl. Screening for the presence of Cre recombinase was performed using primers Cre-forward (TGC CAC GAC CAA GTG ACA GCA ATG) and Cre-reverse (ACCA GAGA CGG AAA TCC ATC GCT C) [Cre band: 650 base pairs (bp)]. Screening for the LSL-tAgo2 at the *Gt(ROSA)26Sor* locus was performed using primers common-forward (CCA AAG TCG CTC TGA GTT GTT ATC), mutant-reverse (CGG GCC ATT TAC CGT AAG), and WT-R (GAG CGG GAG AAA TGG ATA TG). Screening for the presence or absence of the WT allele at the *Lin28a* locus was performed using primers Lin28aRec-F9 (TTC CAG TGG CTT GTA CACC G) and Lin28aRec-R (AAT ACA ACC TTA GTT TCT GCC) [recombined allele: 329 bp; WT allele: 821 bp; loxp band: 1000 bp]. The following thermocycler settings were used: 95°C for 5 min, 35 cycles composed of 30 s at 95°C, 30 s at 61°C, and 1 min at 72°C, followed by 72°C for 10 min.

#### Digital PCR

Digital PCR (QIAcuity digital PCR system, Qiagen; Hopkins Genetic Resources Core Facility) was (*151*, *152*) performed on both DRG and sciatic nerve samples using the following test probes (multiplexed Lin28b Assay ID: Mm01190673_m1; Lin28A Assay ID: Mm00524077_m1) with normalization to an average of the following multiplexed probes (Actin Assay ID: Mm01205647_g1; GAPDH Assay ID: Mm99999915_g1). MicroPartitioning, thermocycling, and fluorescence signal processing were carried out (QIAcuity Software Suite Version 1.2), with quantification of target molecules per microliter determined by multiplying target copies per partition (based on the Poisson distribution) by the number of valid partitions in each well. Sample concentrations were calculated with reference to the partition volume. All samples were tested in triplicate.

#### Nerve injury

SNI was performed as previously described (*55*). Mice were anesthetized with isoflurane (2.5% for induction and maintenance), the left sciatic nerve of 7- to 8-week old mice was exposed at thigh level. The nerve was carefully freed from surrounding connective tissues, near its trifurcation. Tibial and common peroneal nerve branches were ligated, and a small section immediately distal to the ligation was excised. The sural nerve branch was left intact, avoiding contact or stretching. Muscle and skin were sutured with silk 6-0 and 4-0 sutures, respectively.

SNT was performed as previously described (*52*). Mice were anesthetized with isoflurane (2.5% for induction and maintenance), and the left sciatic nerve of 7- to 8-week old mice was exposed at thigh level. The nerve was carefully freed from surrounding connective tissues, near its trifurcation, and was firmly ligated at two locations ∼1 cm apart. The nerve was then completely transected between the pair of ligatures, and a small section of the nerve was removed to prevent rejoining. Muscle and skin were sutured with silk 6-0 and 4-0 sutures, respectively.

#### DRG culture and calcium imaging

DRG cultures were prepared essentially as previously described (*153*), with minor modifications, from 4-week-old Lin28a^flox/−^/Nav1.8^Cre+^ or Lin28a^flox/−^/Nav1.8^Cre−^ mice. Briefly, after harvest and rinsing with complete saline solution (CSS; containing 137 mM NaCl, 5.3 mM KCl, 10 mM glucose, and 10 mM Hepes, with pH adjusted to 7.2 to 7.4 and osmolarity adjusted to 290 to 300 mosmol with mannitol), DRGs were digested first at 37°C in Liberase (Roche Diagnostics) in CSS and then at 37°C in Liberase TM (Roche Diagnostics) supplemented with papain (20 to 30 U/ml; Worthington). Digested ganglia were resuspended in Neurobasal medium supplemented with B27, 1% penicillin/streptomycin, and 5% bovine serum albumin, manually triturated using a pipet tip, passed through a 70-μm filter, plated on glass coverslips that had been coated with poly-d-lysine (Sigma-Aldrich) and laminin (Thermo Fisher Scientific), allowed to adhere, then flooded with medium (Neurobasal/B27/penicillin/streptomycin), and incubated at 37°C with 5% CO_2_. Ratiometric Fura-2 calcium imaging was performed 2 days after plating, essentially as previously described (*154*). Cells were loaded with Fura-2-AM (10 μM) in calcium imaging buffer (CIB; containing 130 mM NaCl, 3 mM KCl, 2.5 mM CaCl_2_·2H_2_O, 0.6 mM MgCl_2_, 10 mM Hepes, 1.2 mM NaHCO_3_, and 10 mM glucose, pH adjusted to 7.45 and osmolarity adjusted to 290 to 300 mosmol with mannitol), and then subjected to Fura-2 ratiometric imaging. To prepare 15 mM KCl and 50 mM KCl solutions, KCl concentration was increased to the desired level and NaCl was proportionately reduced to maintain overall ionic strength. Image pairs were continually acquired at 340-nm 100-ms excitation and 380-nm 200-ms excitation at 1.5-s intervals using an inverted fluorescence microscope (TE200, Nikon) equipped with a complementary metal-oxide semiconductor camera (Neo, Andor) and an automated filter wheel (Lambda), controlled by the NIS elements software (Nikon), and emission at 340/380 nm was calculated. Cells were imaged during continuous perfusion with CIB and then challenged with sequential 30-s applications of 15 mM KCl, capsaicin (10 nM; Sigma-Aldrich), and 50 mM KCl, with 3-min intervening washouts with CIB. Analysis of Fura-2 data was conducted using NIS Elements and Excel (Microsoft) software. Only cells < 1250 μm^2^ were analyzed to enrich for the NaV1.8-expressing population. Cells < 100 μm^2^ were considered too small to be neurons and excluded. Cells with a basal Fura-2 ratio of >0.7 before stimulation were presumed unhealthy and excluded from analysis, as were any cells that failed to exhibit an increase in a Fura-2 ratio of >0.1 in response to either capsaicin or 50 mM KCl. Data were obtained from four coverslips per mouse and four mice (two males and two females) per genotype. After exclusions, data from 199 ± 17 (mean ± SEM) neurons per coverslip were analyzed. Baseline Fura-2 ratio and maximal changes from prestimulus baseline in Fura-2 ratio evoked by KCl or capsaicin were quantified. For coverslip-level analysis, values for all neurons on a given coverslip were averaged and averages compared across 16 coverslips per genotype. For mouse-level analysis, mean values obtained from each coverslip were averaged across four mice per genotype.

#### In situ hybridization

At 3, 7, and 28 days post–SNI surgery, age- and sex-matched mice were transcardially perfused with RNase-free phosphate-buffered saline (PBS) followed by 4% paraformaldehyde in PBS. L3 and L4 DRGs, sciatic nerves, and L3 to L5 spinal cords were removed and immediately postfixed in 4% paraformaldehyde in PBS overnight at 4°C. Tissues were cryoprotected in 30% sucrose for an additional 24 to 48 hours at 4°C, submerged in optimal cutting temperature compound (O.C.T., Tissue-Tek #4583) and flash-frozen. Tissues were cryosectioned and thaw mounted as follows: 12-μm DRG sections were thaw mounted on Superfrost Plus microscope slides (Fisherbrand, #22-034-979), and 12-μm spinal cord transverse sections and 12-μm sagittal sections of sciatic nerves were thaw mounted on poly-d-lysine adhesion coated slides (EMS, #63410) to avoid tissue detachment.

Sections were briefly rinsed with PBS, incubated with RNAscope hydrogen peroxide to quench endogenous peroxidase (ACDBio, #322381) for 10 min at room temperature, and permeabilized with RNAscope protease III (ACDBio, #322381) at 40°C for 30 min. Sections were hybridized with the following RNAscope probes individually at 40°C for 2 hours. DRG sections: Mm-Lin28a (ACDBio, #437121), Mm-Lin28b (ACDBio, #447881), Mm-Fos (ACDBio, #316921), and Mm-Egr1 (ACDBio, #423371). Sciatic nerve sections: Mm-Lin28a and Mm-Lin28b. Spinal cord sections: Mm-Fos and Mm-Egr1. Probe signals were amplified using sequential hybridization with RNAscope Multiplex FL v2 AMP1, AMP2, and AMP3 solutions (ACDBio, #323110), each incubated at 40°C for 30 min. Sequential hybridization of RNAscope Multiplex FL v2 HRP solutions (ACDBio, #323110) for 15 min at 40°C and Opal dyes (Opal 570 or 690, 1:750) were used to visualize RNA staining, each incubated at 40°C for 30 min. Individual fluorescence channels were blocked with RNAscope Multiplex FL v2 HRP blocker solution (ACDBio, #323110) at 40°C for 15 min to allow potential probe multiplexing. Details follow the procedure outlined in a published protocol (*56*).

#### Immunostaining

Immunostaining was performed on both freshly cryosectioned tissues and sections immediately following in situ hybridization. Sections were rinsed three times with 0.1% Triton X-100 in PBS (PBST.1), each for 10 min at room temperature, and subsequently blocked and permeabilized in 0.3% Triton X-100 in PBS (PBST.3) containing 10% normal donkey serum (NDS) for 1 hour at room temperature. Sections were stained overnight with the following primary antibodies diluted in PBST.3 containing 1% NDS at 4°C in a humidity chamber.

Sections immediately following in situ hybridization:

DRG: chicken anti-NeuN (Aves Labs, #NUN, 1:200), rabbit anti-ATF3 (NOVUS, #NBP1-85816, 1:500), and goat anti-GFRα2 (Bio-Techne, AF429, 1:200). Sciatic nerve: rabbit anti-MAP2 (Millipore, #Ab5622, 1:500) and mouse anti-S100B (Millipore, #MAB079-1, 1:200). Spinal cord: mouse anti-NeuN (Chemi-Con, #MAB377, 1:200) and guinea pig anti-PKCγ (Cosmo Bio, #PKCγ-GP-Af350, 1:500).

Freshly cryosectioned section for green fluorescent protein (GFP) validation:

DRG: goat anti-GFP (SICGEN, #AB0020-200, 1:500) and chicken anti-NeuN (Aves Labs, #NUN, 1:200). Sciatic nerve: rabbit anti-GFP (Invitrogen, #A-11122, 1:500), chicken anti-MAP2 (GeneTex, #GTX85455, 1:500), and mouse anti-S100B (Millipore, #MAB079-1, 1:200).

Sections were washed three times with PBST.1, each for 10 min, and then incubated for 2 hours at room temperature in a humidity chamber with secondary antibodies: goat anti-chicken Alexa Fluor 488 (Invitrogen, #A11039), donkey anti-rabbit Alexa Fluor 647 (Jackson ImmunoResearch, #711-605-152), donkey anti-rabbit Alexa Fluor 488 (Jackson ImmunoResearch, #711-545-152), goat anti-mouse Alexa Fluor 647 (Invitrogen, A-21235), goat anti-guinea pig Alexa Fluor 488 (Jackson ImmunoResearch, #106-545-003), donkey anti-goat Alexa Fluor 488 (Jackson ImmunoResearch, #705-545-003), goat anti-chicken Alexa Fluor 568 (Invitrogen, #A11041), donkey anti-chicken Cy3 (Jackson ImmunoResearch, #703-165-155), goat anti-mouse DyLight 405 (Invitrogen, #35501BID), and donkey anti-goat Alexa Fluor 647 (Invitrogen, A-21447). Alexa Fluor 488 Tyramide SuperBoost kit and goat anti-rabbit (Invitrogen, B40943) were used to amplify GFP signals in freshly cryosectioned sciatic nerve sections. Sections were washed three times with PBS, each for 10 min at room temperature, counterstained with Hoechst 33342 (Invitrogen, #H1399, 1:500) if applicable, mounted with Prolong Gold antifade mounting medium (Invitrogen, #36930), and coverslipped. Details follow the procedure outlined in a published protocol (*56*).

#### Confocal imaging and image analysis

Following in situ hybridization and immunostaining, sections were imaged on an LSM 800 confocal microscope (Zeiss) using the ZEN Blue software (Zeiss, version 2.3) with excitation at 405, 488, 561, or 640 nm. Confocal images were taken with a 25X (DRG and spinal cord) or 63X (sciatic nerve) objective.

RNA puncta analysis in DRGs: *Z*-stack confocal images of Lin28a/Lin28b/Fos/Egr1 RNAscope in the DRG, colabeled with NeuN to mark neurons and ATF3 to mark the injured cells, were acquired. Discrete and compound (clustered) RNA puncta were analyzed using FISH-quant (*155*). Cell soma and nucleus outlines were drawn using NeuN and Hoechst images, respectively. Sensory neurons were separated by soma diameter as small/medium cells (predominantly nociceptive neurons) and large neurons (proprioceptors and low-threshold mechanoreceptors) using a diameter cutoff of 700 μm^2^, a previously published guideline (*59*). Discrete RNA puncta were analyzed by assessing program calling of puncta boundaries with visualization in a sample condition-blinded manner. An “average punctum” with quantified fluorescence intensity and volume was collected by averaging the intensity at each pixel for all identified puncta. Point spread function was then used in both the *xy* plane and the *z* axis to quantify compound puncta by defining local minimum and maximum intensity based on the average punctum. The number of discrete puncta and compound puncta was added up. Details follow the procedure outlined in a published protocol (*56*). Injured and uninjured cells were compared in a yoked manner (cells that are of similar size on the same image). No more than eight regions of interest (ROIs; 256 x 256 μm^2^) per section and no more than four sections per mouse were quantified for optimal sampling.

RNA puncta analysis in sciatic nerves: *Z*-stack confocal images of Lin28a/Lin28b RNAscope in the sciatic nerve, colabeled with MAP2 in neuronal processes and S100B in Schwann cells, were acquired. Discrete RNA puncta were analyzed using Imaris (7.6.5 Bitplane Inc.). Three-dimensional (3D) virtual masking of surfaces in sciatic nerve tissue was used for resolving intermingled nerve processes and Schwann cells. Virtual spots were generated for mRNA puncta based on the average 3D dimension of a single punctum. Virtual surfaces were generated for MAP2 and S100 with determined threshold, and thresholds were adjusted to fit the compartment boundaries by visualization of an experimenter blinded to condition. Virtual RNA spots were assigned to each cell type based on surface distance and quantified. Details follow the procedure outlined in a published protocol (*56*). No more than eight ROIs (75 x 75 μm^2^) per section and no more than eight sections per mouse were quantified for optimal sampling.

RNA analysis in the spinal cord: Orthogonal projection confocal images of Fos/Egr1 RNAscope in the spinal cord, colabeled with NeuN and PKCγ, were acquired. RNA expression was analyzed using ImageJ/FIJI. ROIs outlining laminae I and II were drawn on the basis of PKCγ, and the number of neurons (NeuN positive) in the ROI was counted automatically with FIJI Analyze Particles. The number of RNAscope-positive neurons in the ROI was counted manually with Cell Counter Plugin. No more than three sections per mouse were quantified for optimal sampling.

GFP analysis in DRGs and sciatic nerves: Orthogonal projections from confocal images of DRG neurons (from tAgo2/Nav1.8^Cre+^ or tAgo2/Nav1.8^Cre−^) stained for eGFP and NeuN were acquired. Cell outlines were drawn using NeuN projections, and total GFP fluorescence intensity per NeuN-positive cell was averaged over cellular area and quantified using ImageJ/FIJI. No more than 20 cells total per mouse (four to five cells per projection and four to five projections per mouse) were quantified; cellular fluorescence was plotted normalized to the average cellular fluorescence intensity of NeuN-positive cells from Nav1.8^Cre−^ mice. Orthogonal projections were acquired from confocal images of sciatic nerves (from tAgo2/Nav1.8^Cre+^ or tAgo2/Nav1.8^Cre−^) stained for eGFP using the Tyramide SuperBoost kit and colabeled with MAP2 for neuronal processes and S100B for Schwann cells. ROIs were drawn on MAP2 confocal projections; GFP levels were quantified by taking the average fluorescence intensity from ROIs using ImageJ/FIJI. No more than 20 ROIs per mouse (four to five ROIs per projection and four to five projections per mouse) were quantified; average fluorescence intensities were plotted normalized to the average fluorescence intensity from Nav1.8^Cre−^ mice.

#### Chimeric RNA-seq

At 3 and 7 days post–SNI surgery, age- and sex-matched mouse L1 to L5 DRGs and sciatic nerves were dissected and ultraviolet (UV) irradiated three times (4000 μJ/cm^2^ x 100) on a perfluoroalkoxy petri dish on ice, flipping tissues between rounds. DRGs were lysed in lysis buffer [1X PBS supplemented with 1% IGEPAL, 0.5% sodium deoxycholate, 0.1% SDS, 1X HALT protease/phosphatase inhibitor, and 0.64 U (160 U/ml) RNaseOUT (Invitrogen, 10777019)] with a pestle homogenizer. Sciatic nerves were lysed using 1.6-mm stainless steel beads in lysis buffer, with two 30-s cycles in a FastPrep-24 5G bead-beating grinder (MP Biomedicals, 116005500). Tissue homogenates were treated with RNase 1 (0.5 U/1 mg protein) and TURBO DNase (10 U) and centrifuged at 16,100*g* for 15 min at 4°C. Supernatants were subjected to immunoprecipitation with 2 μg of anti-myc (9B11, Cell Signaling 2276) antibody in 500 μl of lysis buffer for 2 hours. The 5′ ends of target RNAs were phosphorylated with PNK, and miRNAs and target RNAs were ligated with T4 RNA ligase 1 to form a chimeric RNA. Phosphate groups on the 3′ ends of the chimeric RNAs were removed with FastAP enzyme, and 3′ adapters were ligated to the chimeric RNA with T4 RNA ligase 2/truncated K227Q. RNA-associated proteins were digested with proteinase K solution (1 mg/ml), and chimeric RNAs were isolated with saturated phenol/chloroform/isoamyl alcohol. The 5′ adapters were ligated to the chimeric RNAs with T4 RNA ligase 1. Chimeric RNA libraries were prepared by reverse transcription, PCR amplification, DNA purification, and size selection. Libraries were multiplexed and sequenced by 150-bp paired-end sequencing with a read depth of 61 to 138 million reads per sample (Illumina HiSeq, NovaSeq X Plus), and chimeric RNA-seq data were analyzed using SCRAP (*44*, *45*). Peak calling in SCRAP was used to define high-confidence interactions across the genome supported by at least three chimeric sncRNA:target RNA reads in each DRG sample library and at least two chimeric sncRNA:target RNA reads in each sciatic nerve library. Details follow the procedure outlined in a published protocol (*43*, *46*). CIMERA-seq, sRNA-seq, and RNA-seq raw and processed data are deposited at NCBI GEO record GSE320207.

#### Seahorse cell mito stress assay

Seahorse cell mito stress assays on sciatic nerve segments were performed following a recently described method (*156*). At 7 days post–SNI surgery, mice were euthanized with isoflurane overdose and cervical dislocation. Ipsilateral sciatic nerves along with naive nerves are isolated, each nerve was divided into five 1.5-mm-long segments and labeled as S1 to S5 for the injured segments, with S1 representing the nerve stump or the injury site. S1 was subsequently cut in half longitudinally to prevent OCR saturation and to increase access of miRNA mimics. Nerve segments were transferred into individual wells of a Seahorse XFe96 spheroid microplate (Agilent, #102905-100), which had been precoated with poly-d-lysine (Sigma-Aldrich, #P7280) overnight. Each well contained 175 μl of Seahorse base medium (Agilent, #10334-100) supplemented with 2 mM glutamine (Agilent, #103579-100) and 6 mM d-glucose (Sigma-Aldrich, #G8270). For let-7 mimic treatment experiments, the miRNA working solution for each well was prepared by diluting 0.6 μl of Lipofectamine RNAiMAX (Invitrogen, #13778075) and 4 (or 8) pmol of hsa-let-7c mirVana miRNA mimic (Ambion, #4464066) or the mirVana miRNA mimic negative control (Ambion, #4464058) in 10 μl of Opti-MEM medium (Gibco, #31985062). Sciatic nerve segments were transferred into spheroid microplates containing 100 μl of Seahorse base medium supplemented with 2 mM glutamine, 6 mM d-glucose, and 10 μl of the miRNA working solution. The plates were incubated in a humidified oxygenated incubator with vehicle or miRNA mimics for 1.5 hours. Before the assay, 65 μl of Seahorse base medium were added to each well. OCRs were measured with a Seahorse XFe96 analyzer (Agilent). An assay cycle of 2-min mix and 1-min wait, and a 3-min measure protocol was repeated three times for baseline rates, five times after oligomycin and carbonyl cyanide *p*-trifluoromethoxyphenylhydrazone (FCCP) injection, and nine times after rotenone/antimycin A injection. Injection ports contained oligomycin (20 μl, 25 μM), FCCP (22 μl, 10 μM), and rotenone with antimycin A (25 μl total, 40 μM rotenone and 20 μM antimycin A). ATP-coupled respiration is calculated as a percent of total OCR by taking the value of the oligomycin-sensitive OCR (basal OCR minus oligomycin-resistant OCR) divided by the total basal OCR, multiplied by 100.

#### Statistical analyses

For RNAscope and immunostaining, when comparing only two groups, two-tailed Student’s *t* test, or Welch’s *t* test for unequal variance, was performed for statistical analysis, with correction for multiple comparisons as indicated. When comparing three and more groups, a one-way analysis of variance (ANOVA) or two-way ANOVA was used. For Seahorse cell mito stress assays, a one-way ANOVA was used. All data were presented as means ± SEM, and the criterion for statistical significance was *P* value < 0.05. All statistical analyses were performed using GraphPad Prism 10.
