# Supplementary material for: Peripheral nerve injury alters metabolic function and neuronal excitability through the Lin28/let-7 pathway
Source: Sci Adv. 2026 Oct 16;12(42):eaeh6791. doi: 10.1126/sciadv.aeh6791 (PMC13674489; doi:10.1126/sciadv.aeh6791)
Supplement: Supplementary file 1 [file sciadv.aeh6791_sm.pdf]

Science Advances
AAAS

Supplementary Materials for

Peripheral nerve injury alters metabolic function and neuronal excitability through the Lin28/let-7 pathway

Xinbei Li *et al.*

Corresponding author: Michael J. Caterina, caterina@jhmi.edu; Mollie K. Meffert, mkm@jhmi.edu

Sci. Adv. **12**, eaeh6791 (2026)
DOI: 10.1126/sciadv.aeh6791

This PDF file includes:

Figs. S1 to S7
Table S1

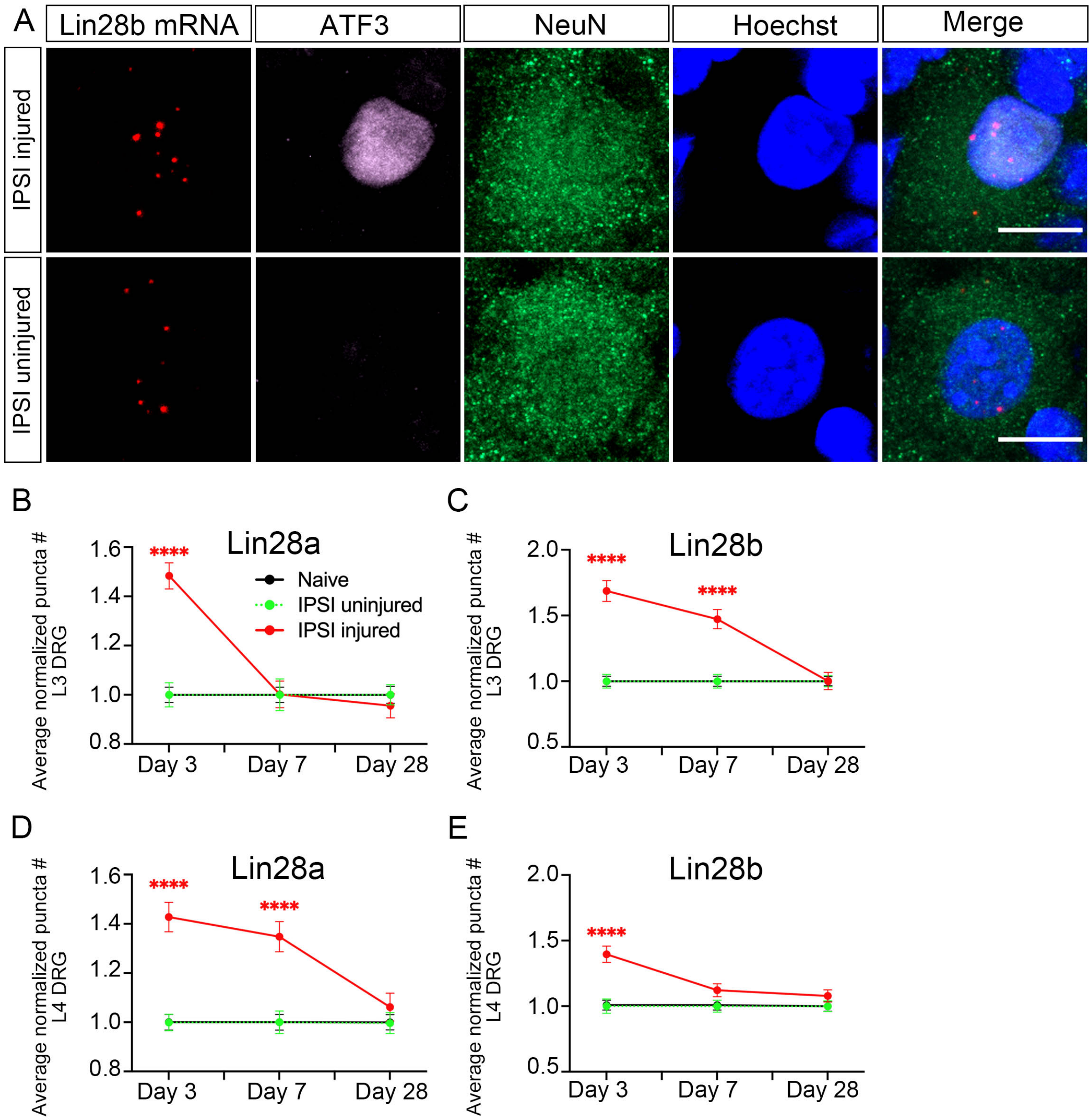

Figure S1:

(A) Representative Lin28b mRNA RNAscope staining in ipsilateral injured and uninjured DRG neurons at 3 dpi (SNI). Neurons are labeled with NeuN, injured neurons are labeled with ATF3, nuclei are labeled with Hoechst. Images are orthogonal projections from 3D Z stack confocal images (Scale bar = 10 μm).

(B and C) Averaged Lin28a (B) and Lin28b (C) mRNA puncta in ipsilateral and naive L3 DRG small to medium sized neurons at 3, 7, 28 dpi (SNI). Number of mRNA puncta in ipsilateral neurons are normalized to uninjured neurons average (set as 1.0) at each time point in a yoked

manner by comparing injured and uninjured neurons within the same image. Number of mRNA puncta in naive neurons are normalized to naive neurons average (set as 1.0). (Statistical comparisons based on mice, two-way ANOVA; Tukey's multiple comparisons test; N = 7 male mice per condition). Error bars represent SEM, ****p < 0.0001.

(D and E) Averaged Lin28a (D) and Lin28b (E) mRNA puncta number in ipsilateral and naive L4 DRG small to medium sized neurons at 3, 7, 28 dpi (SNI). Number of mRNA puncta in ipsilateral neurons is normalized to the uninjured neuron average (set as 1.0) at each time point in a yoked manner by comparing injured and uninjured neurons within the same image. Number of mRNA puncta in naive neurons is normalized to naive neurons average (set as 1.0). (Statistical comparisons based on mice, two-way ANOVA; Tukey's multiple comparisons test; N = 7 male mice per condition). Error bars represent SEM, ****p < 0.0001.

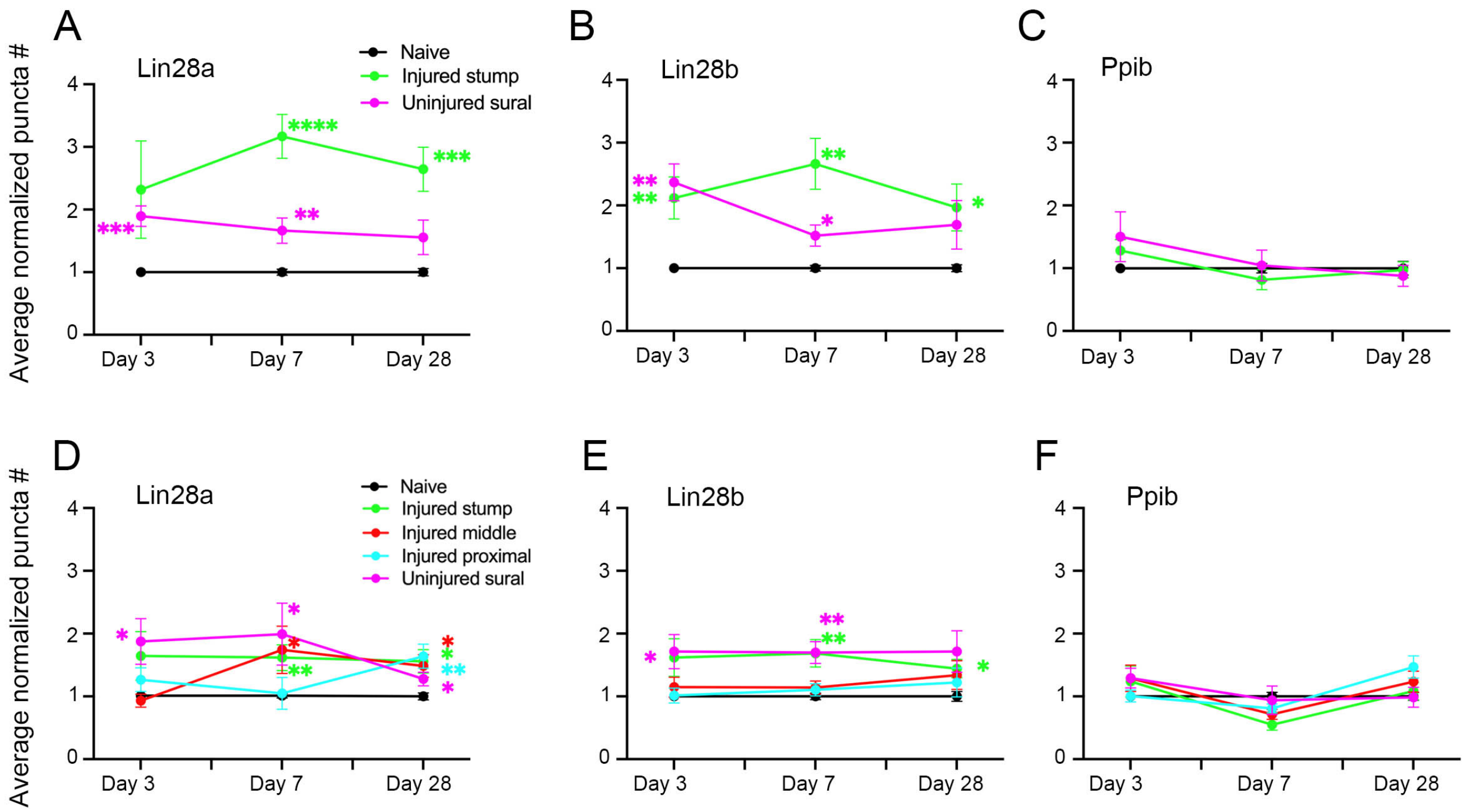

Figure S2:

(A - C) Quantification of Lin28a (A), Lin28b (B), and Ppib (C, positive control) mRNA puncta number in naive and ipsilateral neuronal processes (MAP2+) at 3, 7, 28 dpi (SNI). Number of mRNA puncta in ipsilateral injured nerve stump and uninjured sural nerve are normalized to contralateral uninjured nerves (set as 1.0) at each time point. Number of mRNA puncta in naive nerves are normalized to naive nerves average (set as 1.0). (Statistical comparisons based on regions of interest (ROI), two-way ANOVA; Tukey's multiple comparisons test; N = 40 – 60 ROIs from 7 male mice per condition).

(D - F) Quantification of Lin28a (D), Lin28b (E), and Ppib (F, positive control) mRNA puncta in Schwann cells (S100+) of naive and injured nerve at 3, 7, 28 dpi (SNI). Number of mRNA puncta in ipsilateral injured and uninjured nerves are normalized to contralateral uninjured nerves (set as 1.0) at each time point. Number of mRNA puncta in naive nerves are normalized to the naive nerves average (set as 1.0). (Statistical comparisons based on ROIs, two-way ANOVA; Tukey's multiple comparisons test; N = 40 – 70 ROIs from 7 male mice per condition).

Error bars represent SEM; *$p < 0.05$, **$p < 0.01$, ***$p < 0.001$, ****$p < 0.0001$.

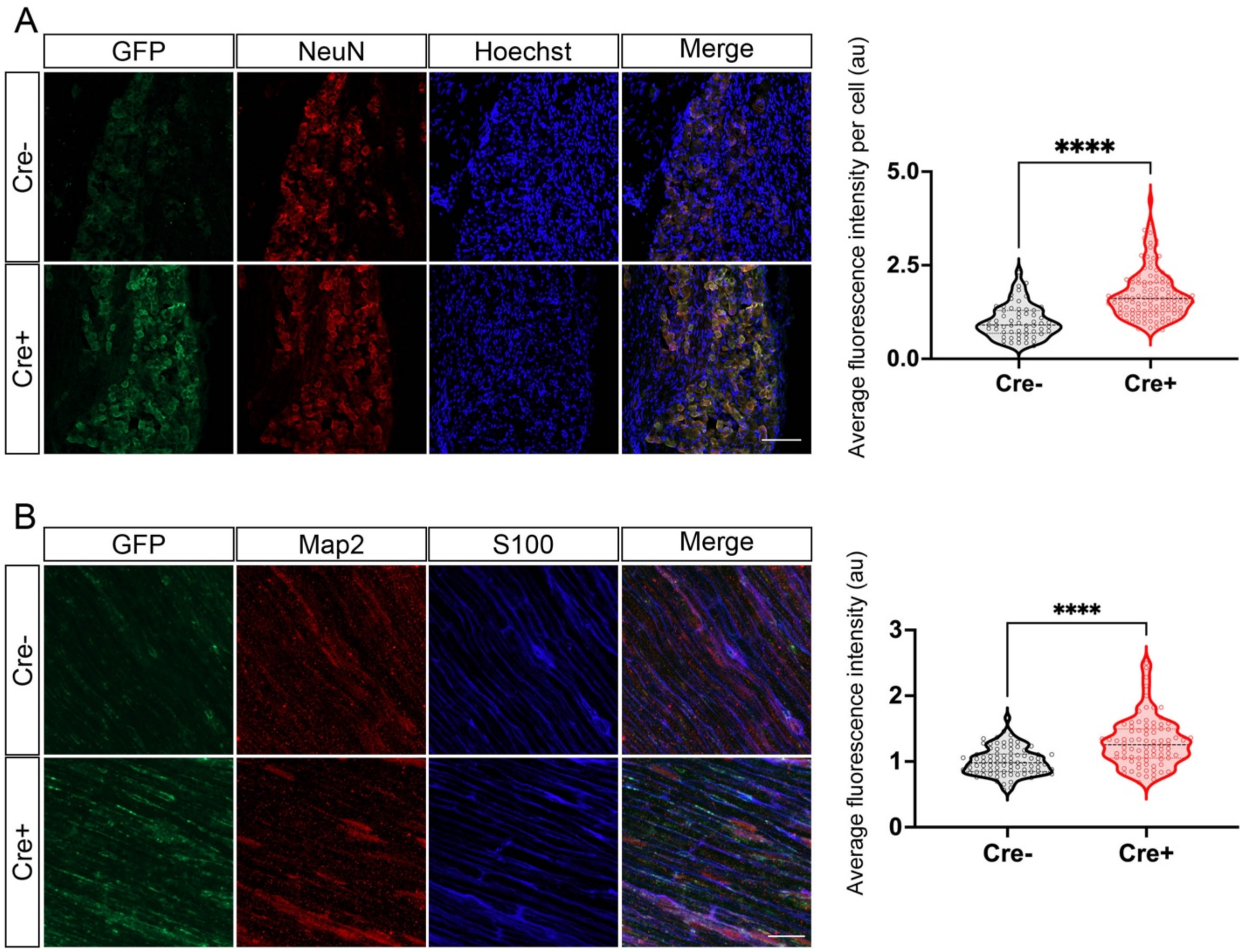

Figure S3:

(A) Representative GFP staining (left, scale bar = 100 µm) and quantification of cellular GFP fluorescence intensity (right) of confocal projections from tAgo2/Nav1.8^{Cre+} or tAgo2/Nav1.8^{Cre-} DRG neurons. Average GFP fluorescence intensities (in arbitrary units (au)) of DRG neurons were normalized to the average fluorescence intensity from tAgo2/Nav1.8^{Cre-} neurons (set as 1.0). Each point is one cell from 4-5 projections per mouse (N = 2 mice of both sexes in tAgo2/Nav1.8^{Cre-}; N = 3 mice of both sexes in tAgo2/Nav1.8^{Cre+}). Two-tailed unpaired Student's *t* test with Welch's correction, ****$p < 0.0001$.

(B) Representative GFP staining (left, scale bar = 20 µm) and quantification of GFP fluorescence intensity (right) of confocal projections from tAgo2/Nav1.8^{Cre+} or tAgo2/Nav1.8^{Cre-} sciatic nerves. Average GFP fluorescence intensities (in au) from ROIs were normalized to the average fluorescence intensity from tAgo2/Nav1.8^{Cre-} (set as 1.0). Each point is one ROI from 4-5

projections per mouse (N = 3 mice of both sexes in tAgo2Nav/1.8^{Cre-}; N = 3 mice of both sexes in tAgo2/Nav1.8^{Cre+}). Two-tailed unpaired Student's *t* test with Welch's correction, ****p < 0.0001.

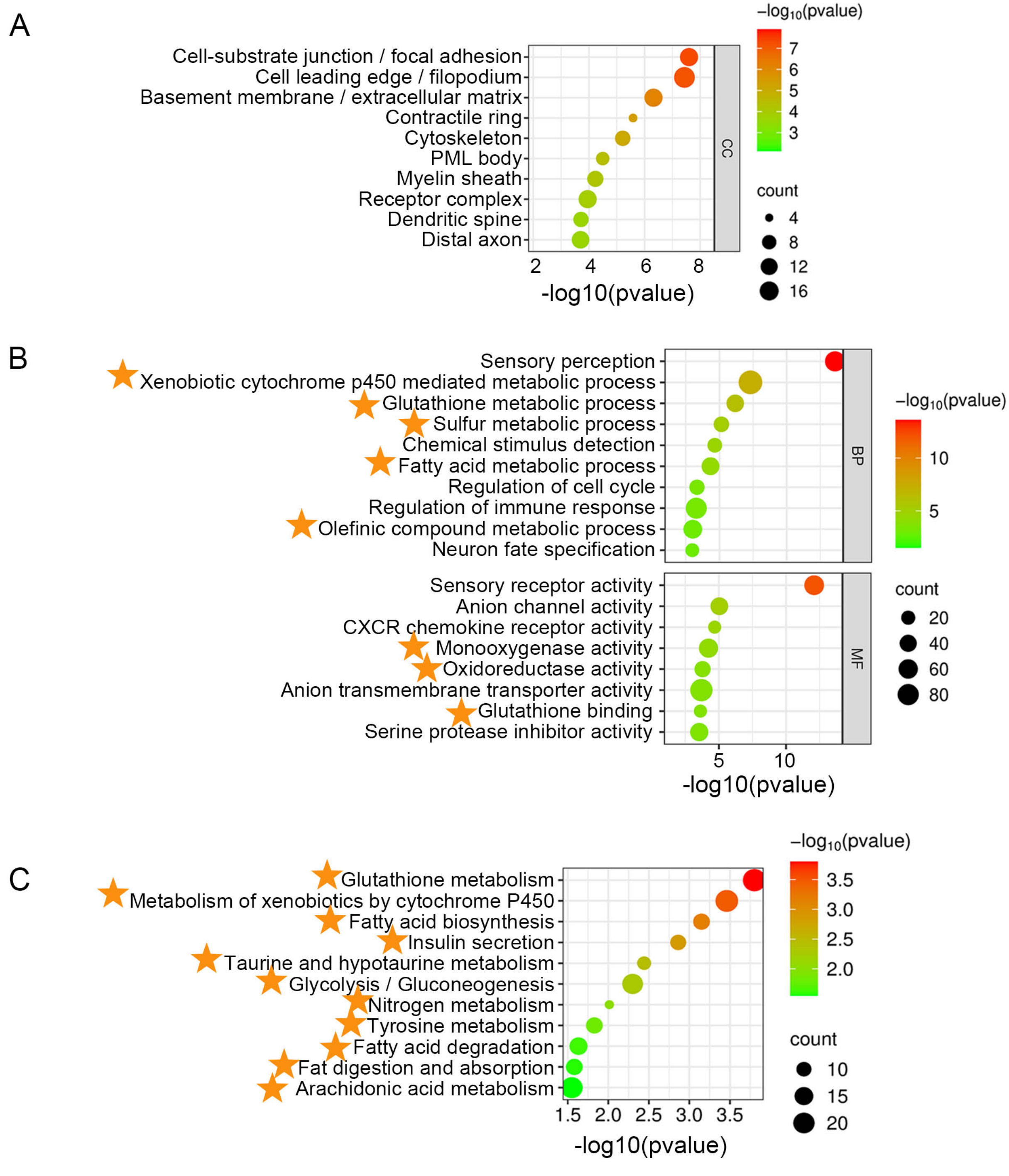

Figure S4:

(A) GO enrichment analysis for cellular components (CC) presenting the top ten GO terms for let-7 gene targets enriched in contralateral sciatic nerve CIMERA-seq compared to ipsilateral sciatic

nerve CIMERA-seq. x axis is enrichment score (-log10(p value)); dot size represents the number of genes; color bar indicates p value. Metabolic related terms are highlighted with stars.

(B) GO enrichment analysis for BP, CC, and MF presenting the top ten GO terms for mRNA transcripts enriched in ipsilateral sciatic nerve bulk RNA-seq compared to contralateral sciatic nerve bulk RNA-seq. x axis is enrichment score (-log10(p value)); dot size represents the number of genes; color bar indicates p value. Metabolic related terms are highlighted with stars.

(C) KEGG enrichment analysis for pathways presenting the top ten KEGG terms for mRNA transcripts enriched in ipsilateral sciatic nerve bulk RNA-seq compared to contralateral sciatic nerve bulk RNA-seq. x axis is enrichment score (-log10(p value)); dot size represents the number of genes; color bar indicates p value. Metabolic related terms are highlighted with stars.

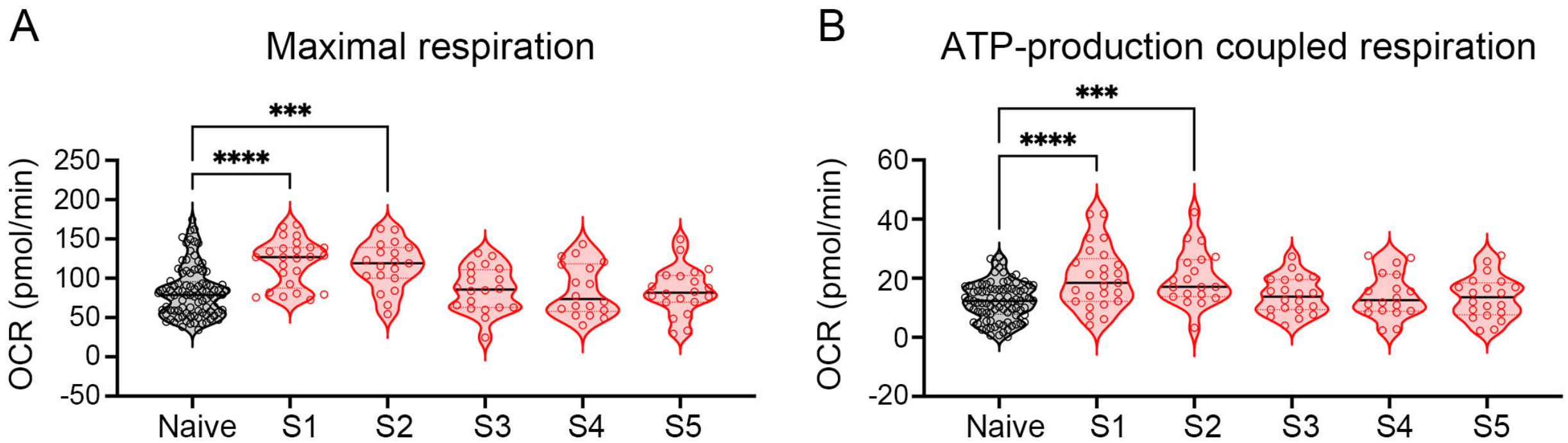

Figure S5:

(A and B) Calculated values for maximal respiration (A) and ATP-production coupled respiration (B) in naive and injured sciatic nerve segments (S1 - S5) at 7 dpi. (one-way ANOVA; Dunnett's multiple comparisons test; N = 18 - 20 male mice per condition). ***$p < 0.001$, ****$p < 0.0001$.

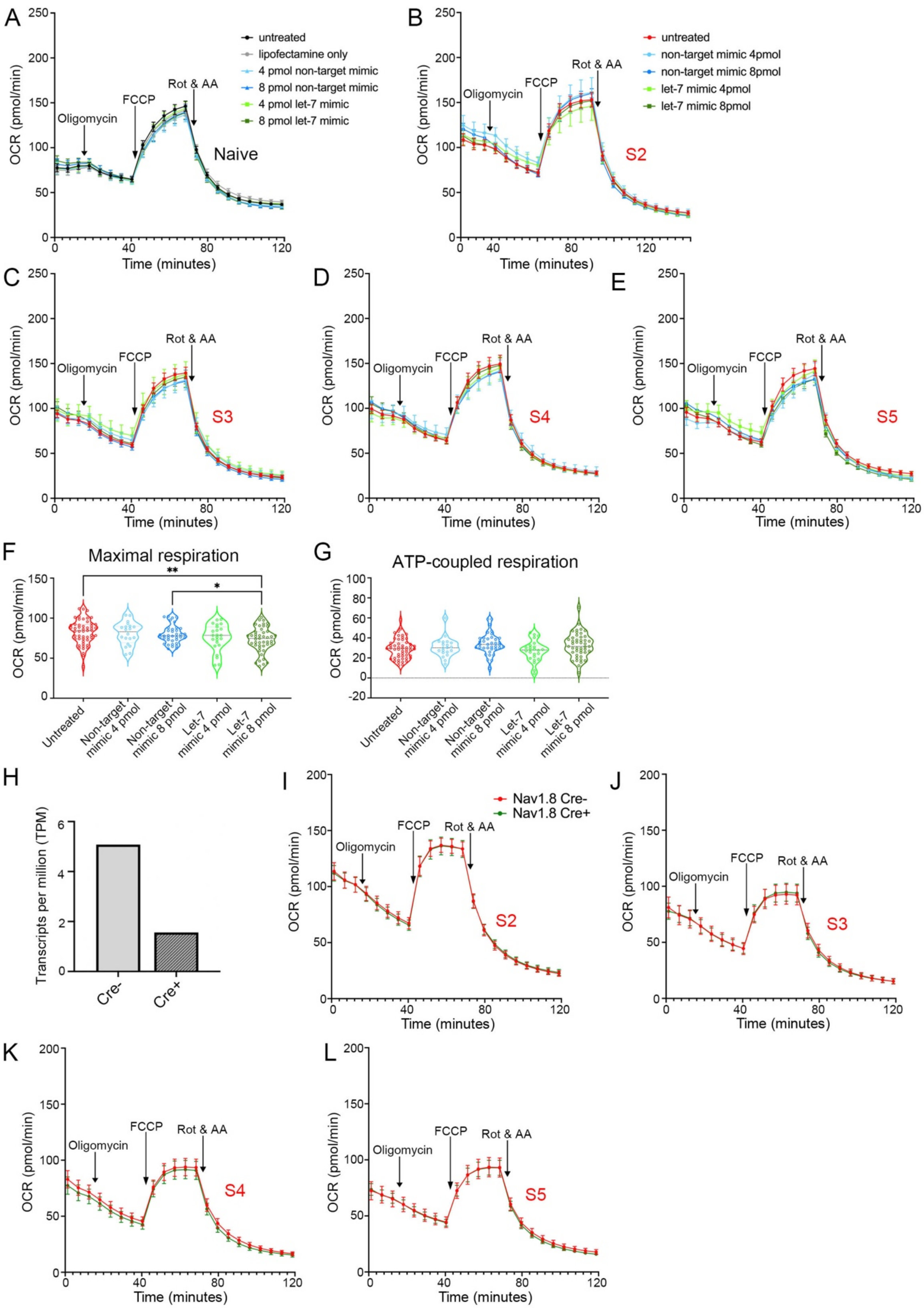

A
untreated
lipofectamine only
4 pmol non-target mimic
8 pmol non-target mimic
4 pmol let-7 mimic
8 pmol let-7 mimic
Oligomycin
FCCP
Rot & AA
Naive
OCR (pmol/min)
Time (minutes)
B
untreated
non-target mimic 4pmol
non-target mimic 8pmol
let-7 mimic 4pmol
let-7 mimic 8pmol
S2
C
S3
D
S4
E
S5
F
Maximal respiration
G
ATP-coupled respiration
Untreated
Non-target mimic 4 pmol
Non-target mimic 8 pmol
Let-7 mimic 4 pmol
Let-7 mimic 8 pmol
H
Transcripts per million (TPM)
Cre-
Cre+
I
Nav1.8 Cre-
Nav1.8 Cre+
J
K
L

Figure S6:

(A) OCR of naive sciatic nerve segments subjects with vehicle only (Lipofectamine RNAiMAX), 4 pmol, 8 pmol of non-target mimic and let-7 mimic in Seahorse Cell Mito Stress Test assay. Each segment was placed into a single well on a 96-well spheroid microplate. (N = 5 - 10 male mice per condition).

(B - E) OCR of injured S2 (B), S3 (C), S4 (D) and S5 (E) at 7 dpi (SNI) subjects with 4 pmol, 8 pmol of non-target mimic and let-7 mimic in Seahorse Cell Mito Stress Test assay. Each segment was placed into a single well on a 96-well spheroid microplate. (N = 12 - 25 male mice per condition).

(F and G) Calculated values for maximal respiration (F) and ATP-production coupled respiration (G) in injured S1 at 7 dpi subjects with 4 pmol, 8 pmol of non-target mimic and let-7 mimic. (one-way ANOVA; Fisher's LSD multiple comparisons test; N = 12 -25 male mice per condition). *p < 0.05, **p < 0.01.

(H) Lin28a transcripts per million (TPM) from bulk sequencing of sciatic nerve from Lin28a$^{flox/-}$/Nav1.8^{Cre-} compared to Lin28a$^{flox/-}$/Nav1.8^{Cre+} mice; expected Cre-mediated reduction but not elimination of Lin28a is observed, as non-Nav1.8 lineage cell types are also present in sciatic nerve. Four sciatic nerves were pooled for each sample.

(I - L) OCR of Lin28a$^{flox/-}$/Nav1.8^{Cre-} and Lin28a$^{flox/-}$/Nav1.8^{Cre+} injured S2 (I), S3 (J), S4 (K), and S5 (L) at 7 dpi (SNI) in Seahorse Cell Mito Stress Test assay. Each segment was placed into a single well on a 96-well spheroid microplate. (N = 20 mice of both sexes in Lin28a$^{flox/-}$/Nav1.8^{Cre-}, N = 19 mice of both sexes in Lin28a$^{flox/-}$/Nav1.8^{Cre+}).

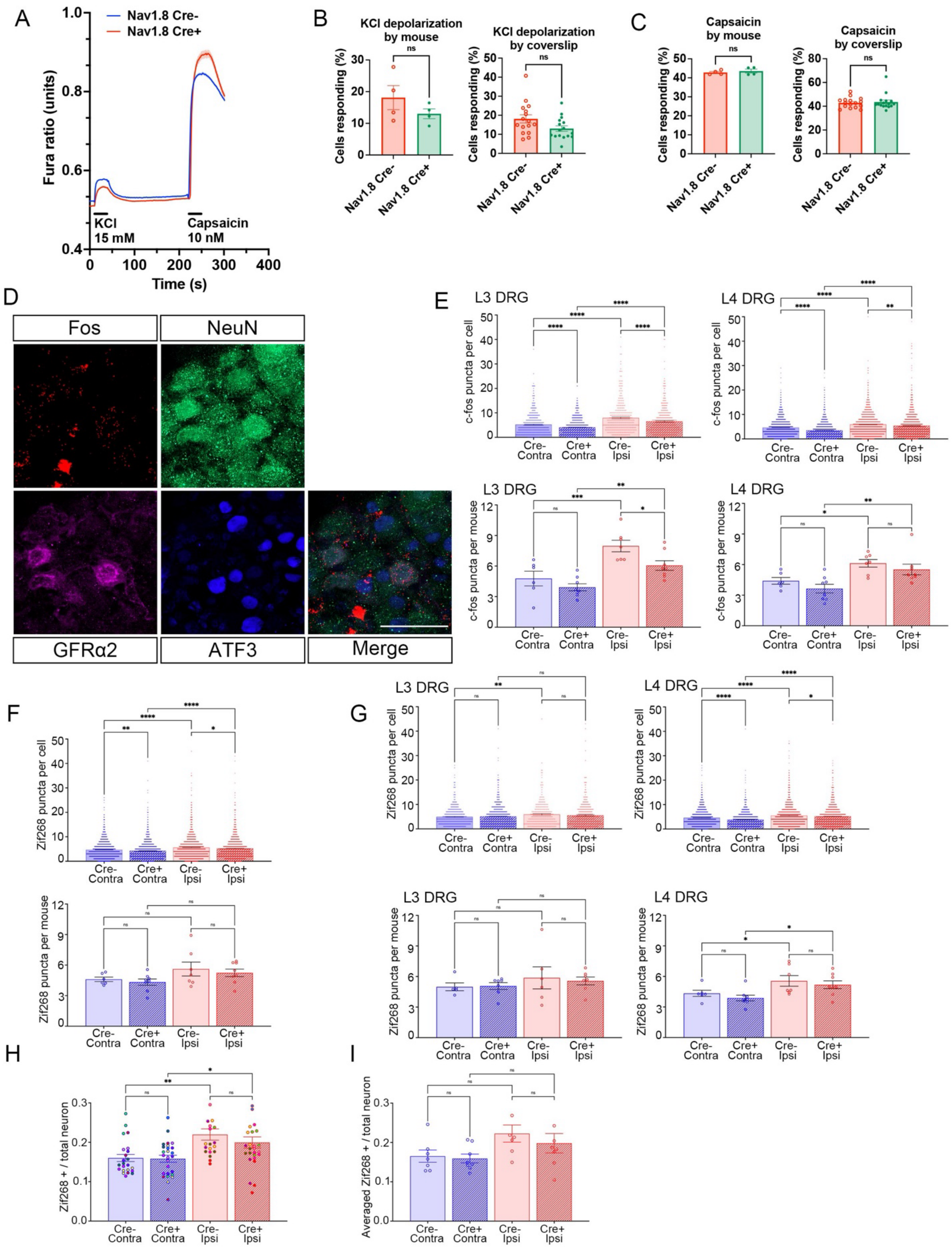
A
Nav1.8 Cre-
Nav1.8 Cre+
Fura ratio (units)
1.0
0.8
0.6
0.4
KCl
15 mM
Capsaicin
10 nM
0
100
200
300
400
Time (s)
B
KCl depolarization
by mouse
KCl depolarization
by coverslip
Cells responding (%)
ns
Nav1.8 Cre-
Nav1.8 Cre+
C
Capsaicin
by mouse
Capsaicin
by coverslip
Cells responding (%)
ns
Nav1.8 Cre-
Nav1.8 Cre+
D
Fos
NeuN
GFRα2
ATF3
Merge
E
L3 DRG
L4 DRG
c-fos puncta per cell
c-fos puncta per mouse
Cre-
Contra
Cre+
Contra
Cre-
Ipsi
Cre+
Ipsi
F
Zif268 puncta per cell
Zif268 puncta per mouse
G
L3 DRG
L4 DRG
Zif268 puncta per cell
Zif268 puncta per mouse
H
Zif268 + / total neuron
I
Averaged Zif268 + / total neuron

Figure S7:

(A-C) Calcium imaging of DRG neurons cultured from Lin28a$^{flox/-}$/Nav1.8^{Cre-} and Lin28a$^{flox/-}$/Nav1.8^{Cre+} mice. (A) Representative traces from Fura-2 ratiometric imaging of neurons during successive stimulation with 15 mM KCl and 10 nM capsaicin. Data shown are Mean ± SEM timecourses across all small diameter neurons from one representative coverslip of each genotype. Blue, Cre-, n=238 neurons; Red, Cre+, n= 104 neurons. (B) Percentage of neurons responding to 15 mM KCl. (C) Percentage of neurons responding to 10 nM capsaicin. Data expressed as mean ± SEM. Symbols represent individual mice (n = 4) in left panels and individual coverslips (n = 16) in right panels. * $p<0.05$, two-tailed, unpaired Welch's t-test.

(D) Representative c-fos mRNA RNAscope staining in ipsilateral Lin28a$^{flox/-}$/Nav1.8^{Cre-} DRG at 7 dpi (SNI). Neurons are labeled with NeuN, nonpeptidergic nociceptive sensory neurons are labeled with GFRα2, injured neurons are labeled with ATF3. Images are orthogonal projections from 3D Z stack confocal images (Scale bar = 50 μm).

(E) Quantification of c-fos mRNA puncta per cell (Top) and averaged c-fos mRNA puncta per mouse (bottom) in L3 (left) and L4 (right) contralateral and ipsilateral GFRα2-positive uninjured neurons in Lin28a$^{flox/-}$/Nav1.8^{Cre-} and Lin28a$^{flox/-}$/Nav1.8^{Cre+} DRG in at 7 dpi (SNI). (one-way ANOVA; Fisher's LSD multiple comparisons test; N = 1000 – 1500 cells from 7 mice of both sexes in Lin28a$^{flox/-}$/Nav1.8^{Cre-}; N = 1000 – 1500 cells from 8 mice of both sexes in Lin28a$^{flox/-}$/Nav1.8^{Cre+}).

(F) Quantification of Zif268 mRNA puncta per cell (top) and averaged Zif268 mRNA puncta per mouse (bottom) in contralateral and ipsilateral GFRa2-positive uninjured neurons in Lin28a$^{flox/-}$/Nav1.8^{Cre-} and Lin28a$^{flox/-}$/Nav1.8^{Cre+} DRG in at 7 dpi (SNI). (one-way ANOVA; Fisher's LSD multiple comparisons test; N = 2000 – 3000 cells from 7 mice of both sexes in Lin28a$^{flox/-}$/Nav1.8^{Cre-}; N = 2000 – 3000 cells from 8 mice of both sexes in Lin28a$^{flox/-}$/Nav1.8^{Cre+}).

(G) Quantification of Zif268 mRNA puncta per cell and averaged Zif268 mRNA puncta per mouse in L3 (left) and L4 (right) contralateral and ipsilateral uninjured neurons in Lin28a$^{flox/-}$/Nav1.8^{Cre-} and Lin28a$^{flox/-}$/Nav1.8^{Cre+} DRG in at 7 dpi (SNI). (one-way ANOVA; Fisher's LSD multiple comparisons test; N = 1000 – 1500 cells from 7 mice of both sexes in Lin28a$^{flox/-}$/Nav1.8^{Cre-}; N = 1000 – 1500 cells from 8 mice of both sexes in Lin28a$^{flox/-}$/Nav1.8^{Cre+}).

(H and I) Quantification of Zif268 positive neurons / total neurons in lamina I and lamina II (H) and averaged c-fos positive neurons / total neurons per mouse (I) in contralateral and ipsilateral spinal cord in Lin28a$^{flox/-}$/Nav1.8^{Cre-} and Lin28a$^{flox/-}$/Nav1.8^{Cre+} DRG in at 7 dpi (SNI). Data points from the same mouse are color-coded consistently (E). (one-way ANOVA; Fisher's LSD multiple comparisons test; N = 30 – 40 spinal cord sections from 7 mice of both sexes in Lin28a$^{flox/-}$/Nav1.8^{Cre-}; N = 30 – 40 spinal cord sections from 8 mice of both sexes in Lin28a$^{flox/-}$/Nav1.8^{Cre+}).

Error bars represent SEM; $^{*}p < 0.05$, $^{**}p < 0.01$, $^{***}p < 0.001$, $^{****}p < 0.0001$.

Table S1: Let-7 metabolic gene targets detected in contralateral and ipsilateral sciatic nerve CIMERA-seq.

Classification of let-7 miRNA-targeted metabolic genes, as categorized by the Mouse Genome Informatics (MGI) Mouse Gene Expression Database. Transcripts encoded by the mitochondrial genome are shown in red and transcripts for which the targeting by let-7 miRNAs was detected in both contralateral and ipsilateral sciatic nerves are shown in bold font.

	Contralateral sciatic nerves				Ipsilateral sciatic nerves			
	Target genes		Reads	Binding partners	Target genes		Reads	Binding partners
**Glucose uptake related genes**	Rps6kb1	ribosomal protein S6 kinase, polypeptide 1	1	let-7a-5p	Myc	myelocytomatosis oncogene	1	let-7b-5p
	Insr	insulin receptor	1	let-7a-5p	Nfe2l2	nuclear factor, erythroid derived 2, like 2	1	let-7i-5p
	Ywhae	tyrosine 3-monooxygenase/tryptophan 5-monooxygenase activation protein, epsilon polypeptide	1	let-7c-5p	Slc2a1	solute carrier family 2 (facilitated glucose transporter), member 1	2	let-7b-5p
**Glycolysis related genes**	Insr	insulin receptor	1	let-7a-5p	Myc	myelocytomatosis oncogene	1	let-7b-5p
	**Plcb1**	**phospholipase C, beta 1**	**2**	**let-7c-5p**	**Plcb1**	**phospholipase C, beta 1**	**2**	**let-7i-5p**
	Pdk2	pyruvate dehydrogenase kinase, isoenzyme 2	1	let-7c-5p				
**Insulin receptor signaling related genes**	Rps6kb1	ribosomal protein S6 kinase, polypeptide 1	1	let-7a-5p	Adipor1	adiponectin receptor 1	2	let-7b-5p
	Pdk2	pyruvate dehydrogenase kinase, isoenzyme 2	1	let-7c-5p				
	Rbm4	RNA binding motif protein 4	1	let-7c-5p				
	Snx5	sorting nexin 5	1	let-7g-5p				
	Igf1r	insulin-like growth factor I receptor	2	let-7c-5p				
	Insr	insulin receptor	1	let-7a-5p				
	Igfbp5	insulin-like growth factor binding protein 5	1	let-7c-5p				
**Mitochondria biogenesis related genes**	Akt3	thymoma viral proto-oncogene 3	1	let-7a-5p	Myc	myelocytomatosis oncogene	1	let-7b-5p
	Stat2	signal transducer and activator of transcription 2	2	let-7c-5p<br>let-7a-5p	Plec	plectin	2	let-7c-5p
	Pum2	pumilio RNA-binding family member 2	2	let-7i-5p	Rnf7	ring finger protein 7	1	let-7g-5p
	Synj2bp	synaptojanin 2 binding protein	2	let-7a-5p				
	Nrf1	nuclear respiratory factor 1	2	let-7b-5p				
	Cep89	centrosomal protein 89	1	let-7c-5p				
	mt-Nd1	mitochondrially encoded NADH dehydrogenase 1	1	let-7c-5p	mt-Cytb	Mitochondrially encoded cytochrome B	1	let-7c-5p
	mt-Nd2	mitochondrially encoded NADH dehydrogenase 2	1	let-7c-5p				
	mt-Nd4	mitochondrially encoded NADH dehydrogenase 4	2	let-7b-5p				
**Mitochondria activity related genes**	**Zfhx3**	**zinc finger homeobox 3**	**1**	**let-7c-5p**	**Zfhx3**	**zinc finger homeobox 3**	**2**	**let-7a-5p**<br>**let-7d-5p**
	Cyp27a1	cytochrome P450, family 27, subfamily a, polypeptide 1	2	let-7c-5p	Stard3	StAR related lipid transfer domain containing 3	1	let-7e-5p
	Cyb5r3	cytochrome b5 reductase 3	4	let-7f-5p	Ern1	endoplasmic reticulum to nucleus signaling 1	1	let-7f-5p
	Canx	calnexin	1	let-7a-5p	Fars2	phenylalanine-tRNA synthetase 2, mitochondrial	2	let-7i-5p
	Rps6kb1	ribosomal protein S6 kinase, polypeptide 1	1	let-7a-5p	Rab11a	RAB11A, member RAS oncogene family	2	Let-7a-5p
	Pdk2	pyruvate dehydrogenase kinase, isoenzyme 2	1	let-7c-5p				
	Synj2bp	synaptojanin 2 binding protein	2	let-7a-5p				
	Sptlc2	serine palmitoyltransferase, long chain base subunit 2	1	let-7f-5p				
	Clybl	citrate lyase beta like	1	let-7d-5p				
	Snn	stannin	1	let-7c-5p				
	Nsun3	NOL1/NOP2/Sun domain family member 3	2	let-7f-5p				
	Gstp1	glutathione S-transferase, pi 1	1	let-7c-5p				
	Fth1	ferritin heavy polypeptide 1	2	let-7a-5p<br>let-7b-5p				
	Ak3	adenylate kinase 3	1	let-7d-5p				
	Ufl1	UFM1 specific ligase 1	1	let-7i-5p				
	Nol6	nucleolar protein family 6 (RNA-associated)	3	let-7f-5p				
	Dnajc30	DnaJ heat shock protein family (Hsp40) member C30	2	let-7c-5p				
	Cep89	centrosomal protein 89	1	let-7c-5p				
	Rab8b	RAB8B, member RAS oncogene family	1	let-7c-5p	mt-Cytb	mitochondrially encoded cytochrome B	1	let-7c-5p
	Lars2	leucyl-tRNA synthetase, mitochondrial	2	let-7c-5p				
	mt-Nd1	mitochondrially encoded NADH dehydrogenase 1	1	let-7c-5p				

	mt-Nd2	mitochondrially encoded NADH dehydrogenase 2	1	let-7c-5p				
	mt-Nd4	mitochondrially encoded NADH dehydrogenase 4	2	let-7b-5p				
**OXPHOS related genes**	Dnajc30	DnaJ heat shock protein family (Hsp40) member C30	2	let-7c-5p	Myc	myelocytomatosis oncogene	1	let-7b-5p
	mt-Nd1	mitochondrially encoded NADH dehydrogenase 1	1	let-7c-5p	mt-Cytb	Mitochondrially encoded cytochrome B	1	let-7c-5p
	mt-Nd2	mitochondrially encoded NADH dehydrogenase 2	1	let-7c-5p				
	mt-Nd4	mitochondrially encoded NADH dehydrogenase 4	2	let-7b-5p				
**TRK/Growth receptor activity related genes**	**Col5a1**	**collagen, type V, alpha 1**	**1**	**let-7i-5p**	**Col5a1**	**collagen, type V, alpha 1**	**2**	**let-7c-5p**
	Col1a1	collagen, type I, alpha 1	1	let-7a-5p	Col3a1	collagen, type III, alpha 1	2	let-7c-5p<br>let-7d-5p
	Itga6	integrin alpha 6	2	let-7f-5p				
	Itgav	integrin alpha V	1	let-7c-5p				
	Igf1r	insulin-like growth factor I receptor	2	let-7c-5p				
	Igfbp5	insulin-like growth factor binding protein 5	1	let-7c-5p				
	Insr	insulin receptor	1	let-7a-5p				
	Fgfr1	fibroblast growth factor receptor 1	1	let-7a-5p				
	Tgfbr3	transforming growth factor, beta receptor III	1	let-7e-5p	Tgfbr1	transforming growth factor, beta receptor I	2	let-7b-5p
					Tgfbr2	transforming growth factor, beta receptor II	1	let-7a-5p
